# Supplementary material for: Precise estimation of in-depth relatedness in biobank-scale datasets using deepKin
Source: Cell Rep Methods. 2025 May 27;5(6):101053. doi: 10.1016/j.crmeth.2025.101053 (PMC12272256; doi:10.1016/j.crmeth.2025.101053)
Supplement: Document S1. Figures S1–S6, Tables S2–S4, and Methods S1 [file mmc1.pdf]

Cell Reports Methods, Volume *5*

Supplemental information

Precise estimation of in-depth relatedness in biobank-scale datasets using deepKin

Qi-Xin Zhang (张齐心), Dovini Jayasinghe, Zhe Zhang (张哲), Sang Hong Lee, Hai-Ming Xu (徐海明), and Guo-Bo Chen (陈国波)

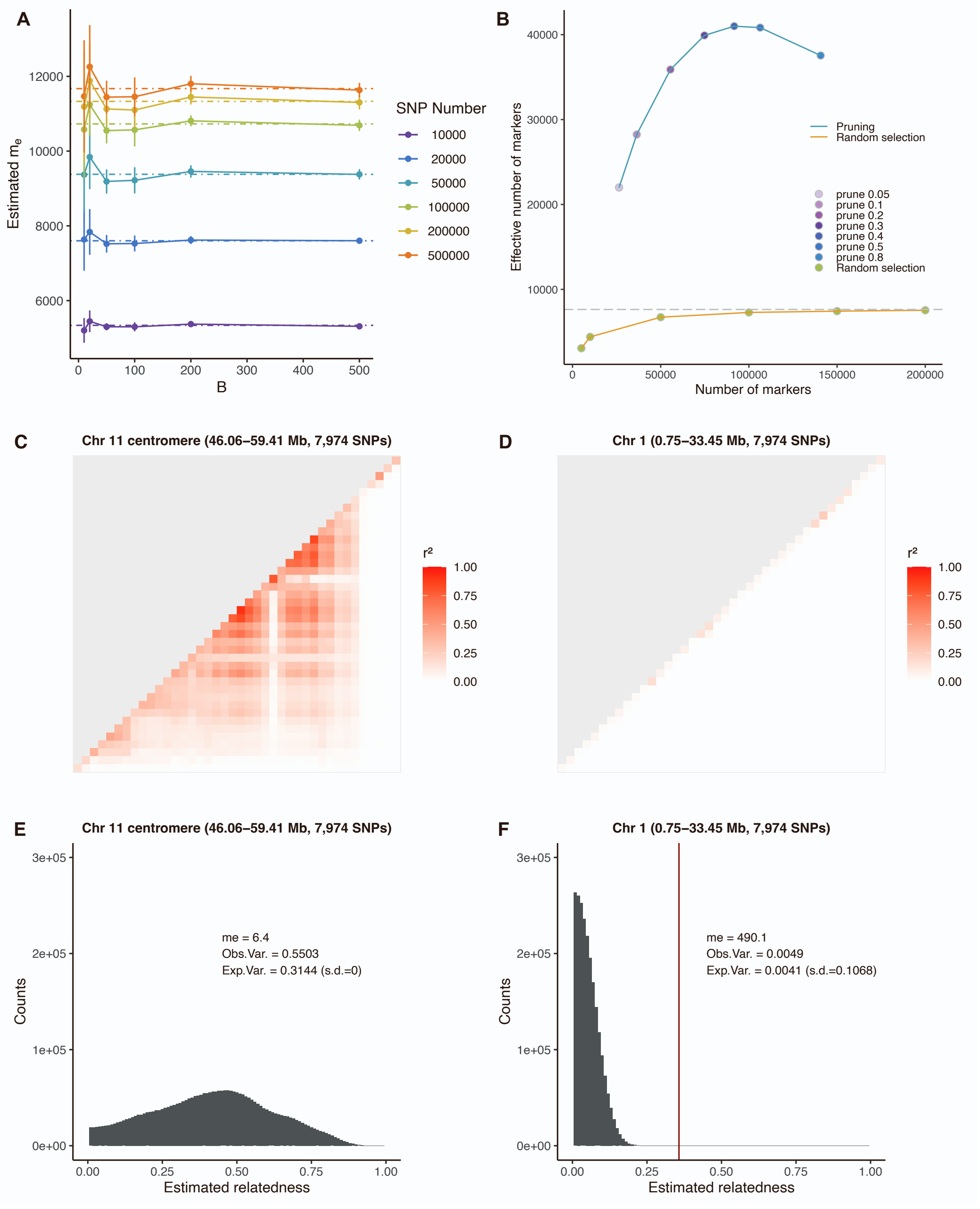

Figure S1 Estimation and evaluation on the effective number of markers (m_e) in 3K Oxford subset, related to STAR methods.

(**A**) The estimated m_e using randomization-based estimator. We have tested on 10K, 20K, 50K, 100K, 200K, and 500K SNPs and the number of iterations from 10 to 500. The dashed lines were the estimated m_e using GRM-based estimator, while each point represented the estimated m_e using randomization-based estimator. As the number of iterations increases, the randomization estimation of m_e approaches the true value.

(**B**) The distribution of the effective number of markers under different SNP sets. A total of 13 SNP sets with different QC criteria were considered, 6 of which were random selected but of different numbers (m =

5,000, 10,000, 50,000, 100,000, 150,000, and 200,000) and 7 were under different pruning thresholds ($r^2 <$ 0.05, 0.1, 0.2, 0.3, 0.4, 0.5, and 0.8). The grey dashed line indicated the m_e of all m = 298,211 variants.

(**C** and **D**) LD heatmap based on two selected genome regions. LD blocks are plotted based on 200 SNP per block.

(**E** and **F**) Histogram plots on estimated relatedness by deepKin based on SNPs from two selected genome regions. Solid line indicates the threshold of significant relatedness given by deepKin.

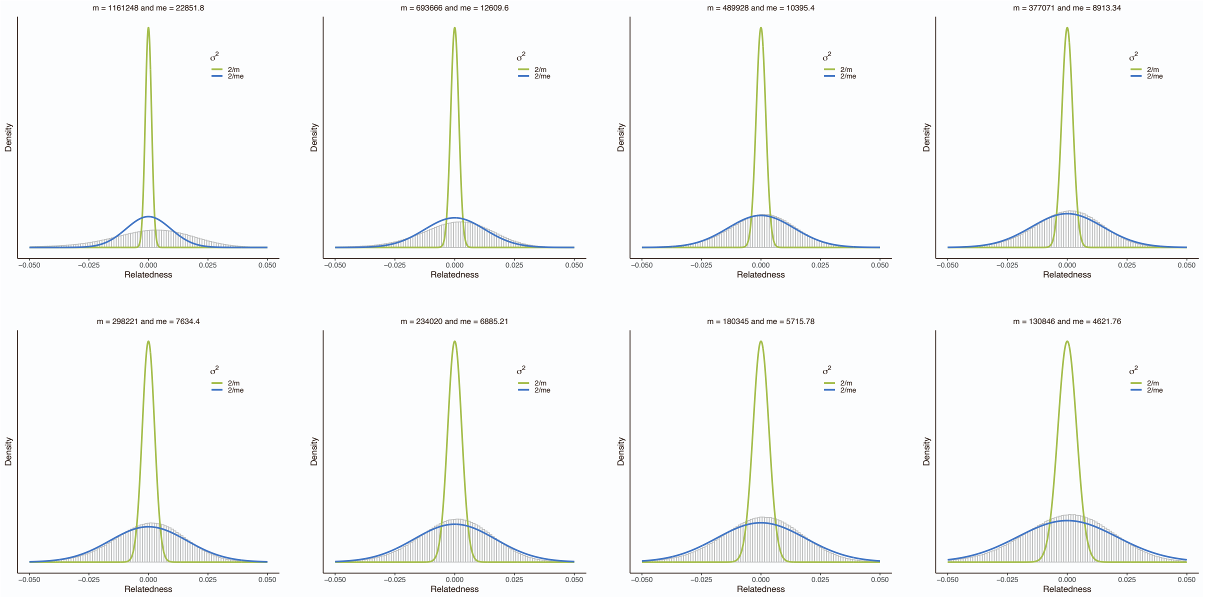

Figure S2 The histogram of estimated relatedness scores based on different MAF thresholds, related to Table 1.

MAF thresholds from left to right, from up to bottom are 0.01, 0.05, 0.10, 0.15, 0.20, 0.25, 0.30, and 0.35. The green and blue curves indicate the normal distribution of $N(0, 2/m)$ and $N(0, 2/m_e)$, respectively.

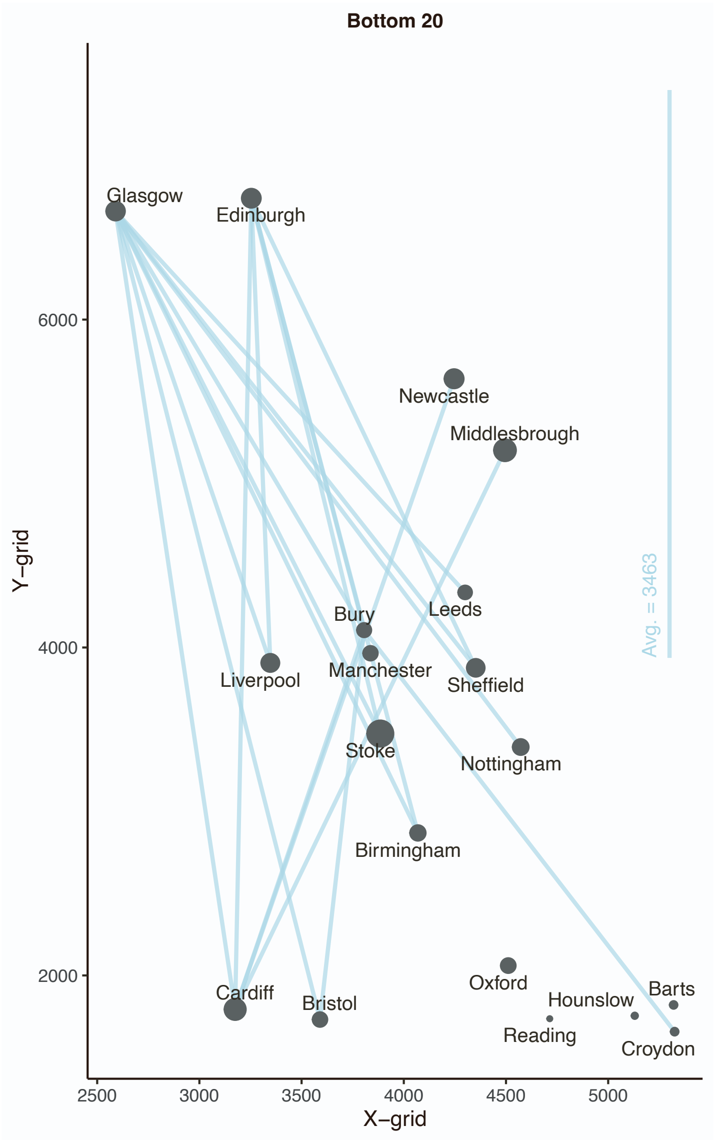

Figure S3 Grid coordinates of 19 assessment centers in UKB and the bottom 20 pair-wise proportion of cross-cohort significant relatives, related to Figure 5.

The averaging distance is calculated from the average straight-line distance of 20 pairs of cohorts in the plot. The size of the dot indicates the size of the proportion of within-cohort significant relatives.

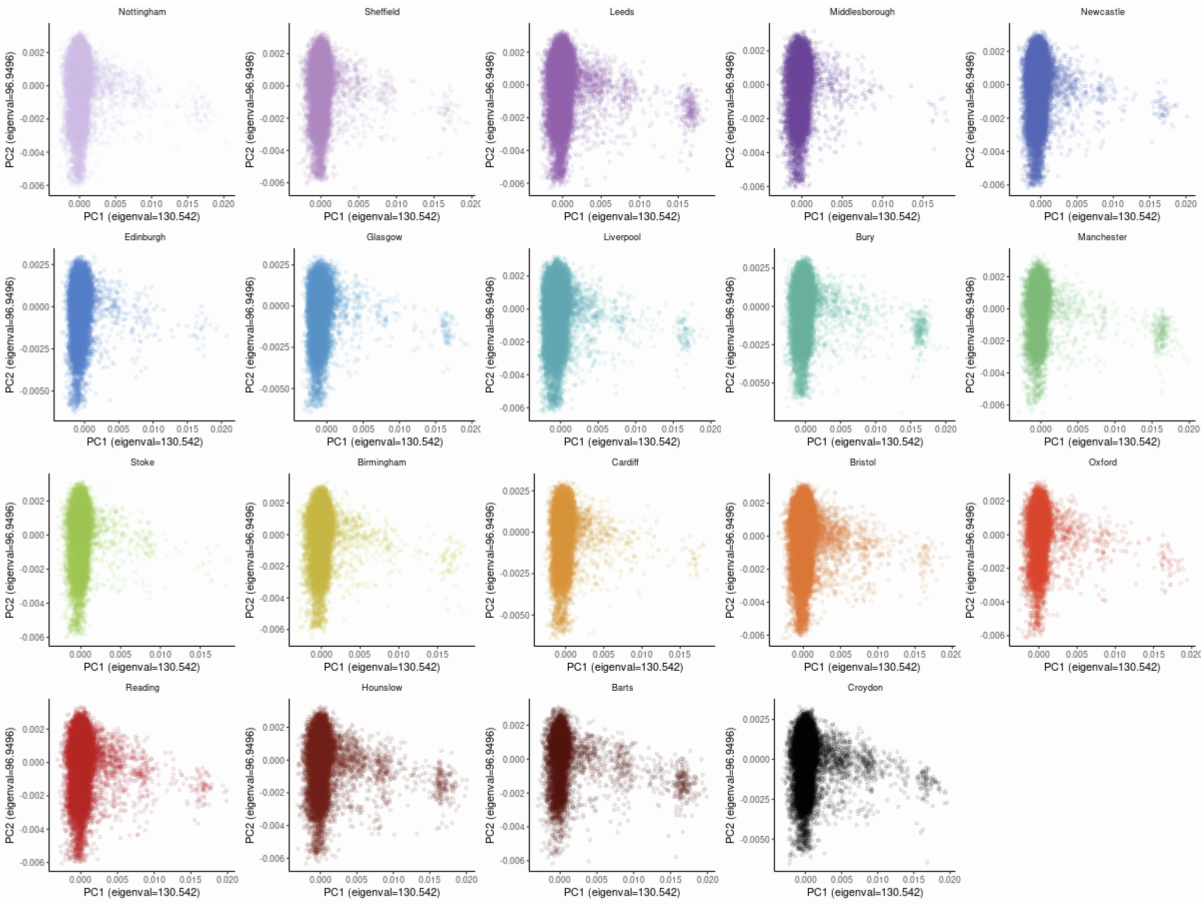

Figure S4 The distribution of the first two PCs for individuals in the 19 UKB cohorts, related to Figure 5.

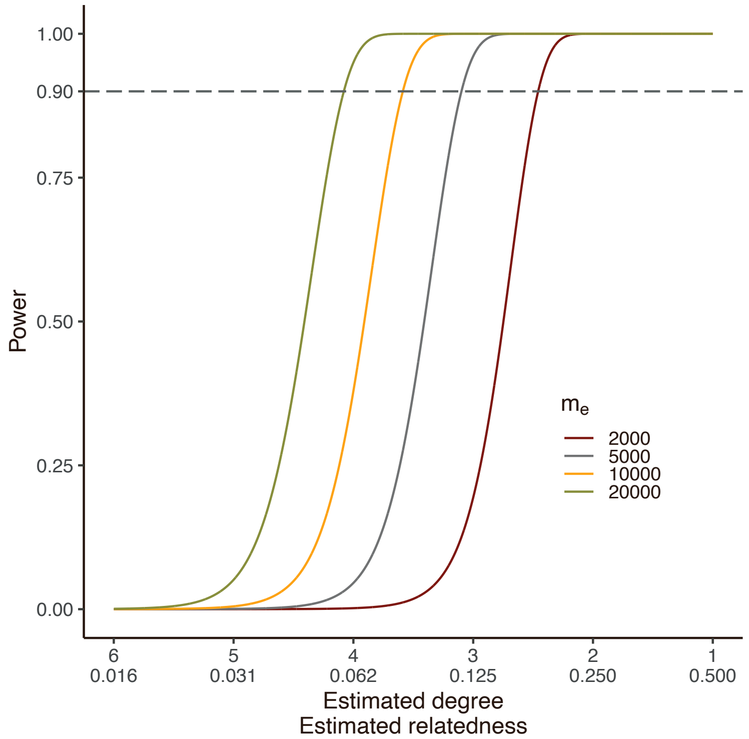

Figure S5 The power of detecting relatedness under different effective number of markers, related to STAR methods. The power is calculated based on Eq 7 under four different effective number of markers (m_e = 2,000, 5,000, 10,000, and 20,000). The dashed grey line indicates 90% power. A range of degrees from zero to six are considered. Suppose Type I error rate of $\alpha = 0.05/N$ and Type II error rate of β=0.1, where $N = 40{,}000$.

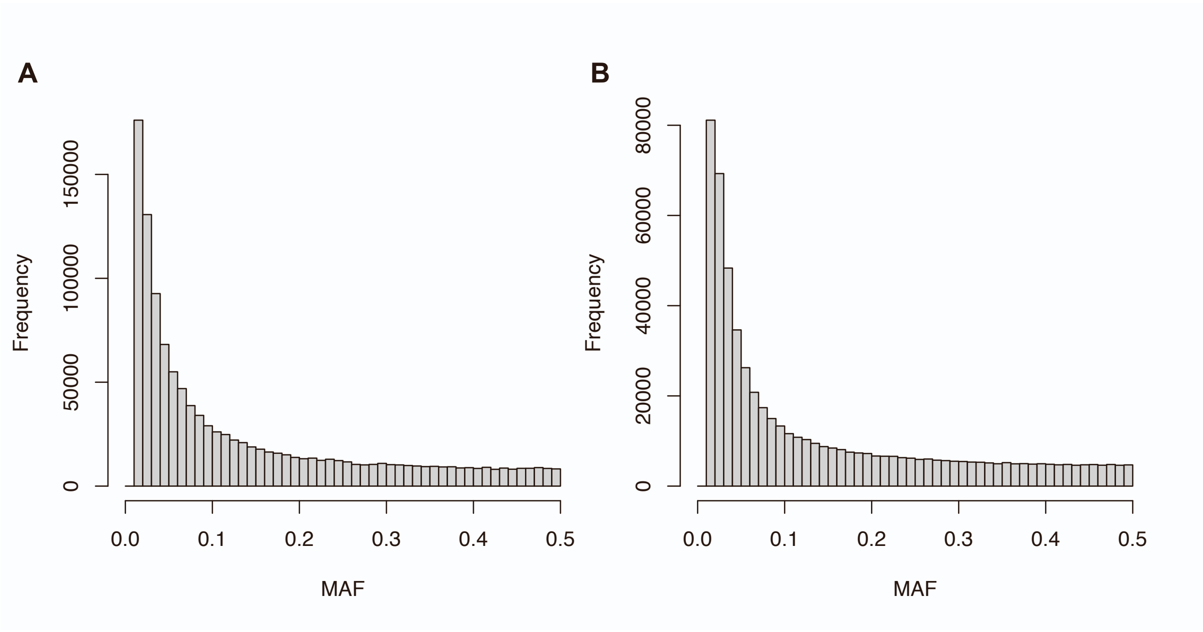

Figure S6 The histograms of MAF distribution in two UKB subsets after QC, related to STAR methods. (**A**) In 3K Oxford subset and (**B**) in 430K white British subset. QC criteria: MAF > 0.01; HWE test *p*-value > 1e-7; no locus missingness.

Table S2 QC details for four SNP sets and the numbers of pairs exceed relatedness cut-offs based on two normal GRM cutoffs in the Oxford demo, related to Table 1.

SNP set	QC details	Number of SNPs	m_e	δ	GRM Cut-off	Number of pairs
Set 1	MAF>0.05	693,666	12,610	3.827	θ>0.05	2,381
					θ>0.025	47,268
Set 2	MAF>0.2	298,221	7,634	3.465	θ>0.05	539
					θ>0.025	72,032
Set 3	MAF>0.2;	237,642	10,424	3.690	θ>0.05	108
	eigenGWAS *p*-value>0.05				θ>0.025	22,564
Set 4	MAF>0.2;	36,425	28,244	4.409	θ>0.05	60
	LD pruning (50 5 0.1)				θ>0.025	655

Table notes: 3,000 individuals and a total number of ~4.5 million comparisons are considered.

*δ is the deepest significant degree that the data would support to detect from unrelated individuals.

*m_e is estimated by randomized estimation.

Table S3 Summary of the significant related pairs in UKB white British dataset, related to Figure 5.

Degree	Identical	1st-degree	2nd-degree	3rd-degree	4th-degree	5th-degree	Total
Number of pairs	162	25,699	9,455	53,221	91,977	31,606	212,120

Table S4 Real dataset examples for applying the deepKin guidelines, related to STAR methods.

Data set	n	m_e (m)	Guideline I		Guideline II	
			θ^t	min m_e	θ^t	Power
UKB white British	427,287	56,945 (72,016)	1	101	1	1.000
			0.5	482	0.5	1.000
			0.25	2,089	0.25	1.000
			0.125	8,690	0.125	1.000
			0.0625	35,437	0.0625	1.000
			0.03125	143,116	0.03125	0.028
UKB Chinese	1,435	38,948 (238,789)	1	57	1	1.000
			0.5	285	0.5	1.000
			0.25	1,267	0.25	1.000
			0.125	5,331	0.125	1.000
			0.0625	21,855	0.0625	1.000
			0.03125	88,493	0.03125	0.158
Middle East	137	22,049 (1,321,919)	1	39	1	1.000
			0.5	203	0.5	1.000
			0.25	920	0.25	1.000
			0.125	3,905	0.125	1.000
			0.0625	16,075	0.0625	0.989
			0.03125	65,223	0.03125	0.124

Table notes: n is the number of sample size; m is the number of markers; m_e is the effective number of markers and is estimated by randomized estimation. We suppose Type I error rate of $\alpha = 0.05/N$ and Type II error rate of $\beta = 0.1$, where $N = n(n-1)/2$ The first two datasets are from UKB and the third data sets are from https://doi.org/10.1016/j.cell.2021.07.013.

Methods S1. All about m_e, related to STAR Methods

Contents

1 Introduction **2**

2 Definition of m_e **2**

3 Estimation for m_e **3**

3.1 Method I: GRM-based estimation 3

3.2 Method II: $tr(\mathbf{K}^2)$ estimation 4

4 General linkage disequilibrium and m_e **5**

1 Introduction

In both the linkage (family-based design) and the GWAS (population-based design) era, the concept of m_e is raised and developed. The concept of "independent number of segments/markers" raises in particular when genome-wide markers are involved in determining the sampling variance of population statistics, such as the sampling variance of multilocus identity by descent (IBD) score in terms of chromosomal length $m_{e.L}$. The early pioneering work relevant to $m_{e.L}$ can be found in Stam, who estimated multilocus IBD (1), and a good example of how the chromosomal length that determines the distribution of IBD for full sibs can be found in Visscher et al. (2); Hill and Weir gave a comprehensive summary of how $m_{e.L}$ determines the sampling variance for various relatives (3). If $m_{e.L}$ is related to chromosomal recombination, while in the GWAS era m_e is related to linkage disequilibrium (LD). From genomic selection (4; 5; 6), polygenic risk score (7; 8; 9), estimation of the sampling variance of SNP-heritability (h^2_{SNP}) (10; 11; 12; 13), and even in the emerging field such as differential privacy for membership inference (14), m_e is deeply involved.

2 Definition of m_e

As a continuous concept from the linkage to the GWAS era like m_e, we nevertheless only focus on its implication in GWAS here. The m_e naturally creeps in when the genetic relationship matrix (GRM) is involved. We give some typical circumstances that m_e shows in

$$\begin{cases} var(h^2_{snp}) & = \frac{2m_e}{n^2} \quad (10;\ 15) \\ var(\theta) & = \frac{1+\theta^2}{m_e} \quad (14) \end{cases} \tag{1}$$

$var(h^2_{SNP})$ is the sampling variance for h^2_{SNP} under REML, but it is variations of this term when the method is switched to method of moment (MoM) (11; 13). Even under its randomized application for MoM, the sampling variance the estimated h^2_{SNP} involved m_e but with further modification (16). θ is realized genetic relatedness estimated in genome-wide markers.

For a dataset of n individuals and m SNPs, GRM is defined as

$$\mathbf{K} = \frac{\mathbf{X}\mathbf{X}^T}{m} \tag{2}$$

and $\theta_{i,j}$ refers to the ith and j^{th} element in $\mathbf{K}$,

$$\theta_{i,j} = \frac{1}{m}\sum_{l}^{m} x_{i,l}x_{j,l} \tag{3}$$

A global measure of LD for m^2 pairs of markers is

$$m_e = \frac{m^2}{\sum_{l_1,l_2} r^2_{l_1l_2}} = \frac{m^2}{m + \sum_{l_1\neq l_2} r^2_{l_1l_2}} \tag{4}$$

in which $r^2_{l_1l_2}$ the squared correlation between a pair of biallelic loci l_1 and l_2 (17). Furthermore, $r^2_{l_1l_2} =$

$\frac{D^2_{l_1l_2}}{p_{l_1}q_{l_1}p_{l_2}q_{l_2}}$, in which $D_{l_1l_2}$ is another commonly used LD metric and $q_{l_1} = 1 - p_{l_1}$ (p_{l_1} the allele frequency of the l_1^{th} locus). Obviously, when all markers are in pairwise linkage equilibrium, $m_e = m$ because of $\sum_{l_1 \neq l_2} r^2_{l_1l_2} = 0$. According to the Isserlis theorem (18), we have

$$\begin{cases} E(\theta_{ij,l_1}\theta_{ij,l_2}) &= E(x_{il_1}x_{jl_1})E(x_{il_2}x_{jl_2}) + E(x_{il_1}x_{il_2})E(x_{jl_1}x_{jl_2}) + E(x_{il_1}x_{jl_2})E(x_{jl_1}x_{il_2}) \\ &= \theta_{ij}r_{l_1l_1}\theta_{ij}r_{l_2l_2} + \theta_{ii}\theta_{jj}r^2_{l_1l_2} + \theta^2_{ij}r^2_{l_1l_2} = \theta^2_{ij} + (\theta_{ii}\theta_{jj} + \theta^2_{ij})r^2_{l_1l_2} \\ E(\theta_{ij,l_1}) &= \theta_{ij}r_{l_1} \\ E(\theta_{ij,l_2}) &= \theta_{ij}r_{l_2} \end{cases} \tag{5}$$

One kernel element is

$$\begin{cases} cov(\theta_{ij,l_1}, \theta_{ij,l_2}) &= E(\theta_{ij,l_1}\theta_{ij,l_2}) - E(\theta_{ij,l_1})E(\theta_{ij,l_2}) \\ &= (\theta_{ij}r_{l_1l_1}\theta_{ij}r_{l_2l_2} + \theta_{ii}\theta_{jj}r^2_{l_1l_2} + \theta^2_{ij}r^2_{l_1l_2}) - \theta_{ij}r_{l_1}\theta_{ij}r_{l_2} \\ &= \theta_{ii}\theta_{jj}r^2_{l_1l_2} + \theta^2_{ij}r^2_{l_1l_2} \\ var(\theta_{ij,l_1}) &= \theta_{ii}\theta_{jj}r^2_{l_1l_1} + \theta^2_{ij}r^2_{l_1l_1} \end{cases} \tag{6}$$

For unrelated individuals, $cov(\theta_{ij,l_1}, \theta_{ij,l_2}) = r^2_{l_1l_2}$ and $var(\theta_{ij}) = 1$; the correlation between θ_{ij,l_1} and θ_{ij,l_2} is of $r^2_{l_1l_2}$.

3 Estimation for m_e

From the first glance, the estimation of m_e has a computational cost of $\mathcal{O}(nm^2)$, which is infeasible if m is, unfortunately the case in GWAS, big. Almost no conventional methods tackle the estimation of m^2 pairwise LD (19; 20). So, a couple of methods have been developed to estimate m_e.

3.1 Method I: GRM-based estimation

In appendix 1 of Goddard's work (4), a method is proposed to estimate global LD for m^2 of SNPs

$$m_{e.1} = \frac{1}{var(K_o)} \tag{7}$$

in which K_o is the $\frac{n(n-1)}{2}$ off-diagonal elements in $\mathbf{K}$. Furthermore, the asymptotic distribution of $m_{e.2}$ is $N(m_e, \frac{4m_e^2}{n^2})$.

So, the mathematical expectation is (for unrelated samples $E(\theta_{ij}) = 0$)

$$\begin{aligned} var(K_o) &= E(\theta^2_{ij}) - E^2(\theta_{ij}) \\ &= \tfrac{1}{m^2}\textstyle\sum^m_{l_1,l_2}(\theta_{ii}\theta_{jj}r^2_{l_1l_2} + \theta^2_{ij}r^2_{l_1l_2}) \\ &= \tfrac{1}{m^2}\textstyle\sum^m_{l_1,l_2} r^2_{l_1l_2} \end{aligned} \tag{8}$$

, and $E(1/var(K_0)) = m_e$ is a unbiased estimate for m_e. It should be noted that the computational cost is

$O(n^2m)$, which will soon be infeasible for a large sample size.

3.2 Method II: $tr(\mathbf{K}^2)$ estimation

Under random mating, we have

$$\begin{aligned}
tr(\mathbf{K}^2) &= \textstyle\sum_{i,j}^{n}\sum_{l_1,l_2}^{m}\theta_{i,j}^2 \\
&= \textstyle\sum_{i,j}^{n}\sum_{l_1,l_2}^{m} x_{i,l_1}x_{i,l_2}x_{i,l_1}x_{j,l_2} \\
&= \textstyle\sum_{i,j}^{n}\{\sum_{l}^{m} x_{i,l}^2 x_{j,l}^2 + \sum_{l_1,l_2}^{m} x_{i,l_1}x_{i,l_2}x_{j,l_1}x_{j,l_2}\} \\
&= \textstyle\sum_{i,j}^{n}\{\sum_{l}^{m}[E(x_{i,l}^2)E(x_{j,l}^2) + 2E^2(x_{i,l}x_{j,l})] \\
&\quad \textstyle+\sum_{l_1\neq l_2}^{m}[E(x_{i,l_1}x_{i,l_2})E(x_{j,l_1}x_{j,l_2}) + E(x_{i,l_1}x_{j,l_1})E(x_{i,l_2}x_{j,l_2}) + E(x_{i,l_1}x_{j,l_2})E(x_{i,l_2}x_{j,l_1})]\} \qquad (9) \\
&= \textstyle\sum_{i,j}^{n}\{\sum_{l}^{m}(\theta_{ii}\theta_{jj}r_{ll}^2 + 2\theta_{ij}^2 r_{ll}^2) \\
&\quad \textstyle+\sum_{l_1\neq l_2}^{m}(\theta_{ii}\theta_{jj}r_{l_1l_2}^2 + \theta_{ij}^2 r_{l_1l_1}r_{l_2l_2} + \theta_{ij}^2 r_{l_1l_2}^2\} \\
&= \textstyle\sum_{i,j}^{n}\{\sum_{l}^{m}(1+2\theta_{ij}^2) + \sum_{l_1\neq l_2}^{m}(r_{l_1l_2}^2 + \theta_{ij}^2 + \theta_{ij}^2 r_{l_1l_2}^2)\} \\
&= n(n+1)\textstyle\sum_{l_1,l_2}^{m} r_{l_1l_2}^2 + n
\end{aligned}$$

For biobank-scale data, $\mathbf{K}$ is so expensive to compute, a novel estimation for m_e is obtained via a randomized algorithm as follows (21)

$$L_B = \frac{1}{Bm^2}\sum_{b=1}^{B}\mathbf{z}_b^T\mathbf{X}\mathbf{X}^T\mathbf{X}\mathbf{X}^T\mathbf{z}_b, \qquad (10)$$

$\{\mathbf{z}_b : b = 1, 2, \cdots, B\}$ are vectors sampled from the standard normal distribution $N(0,1)$, and $E(L_B) = tr(\mathbf{K}^2)$. Thus, the computational time complexity is $O(nmB)$. We have a randomized estimator below

$$\begin{cases} m_{e.2} = \frac{n(n+1)}{L_B - n} \\ \mathrm{var}(m_{e.2}) = \frac{2\hat{m}_e^4}{n^4}\frac{\mathrm{tr}(\mathbf{K}^4)}{B} = \frac{\hat{m}_e^4}{n^4}\sigma_{L_B}^2 \end{cases} \qquad (11)$$

Interestingly, there is a variation for Method II (supplementary page 36 in (7)). This method is a simulation algorithm that evaluates quasi-independent genomic markers in GWAS data in n individuals and m SNPs. The algorithm is described in the following.

1 Randomly assign 0 or 1 to each individual with a probability p of 0.5; it mimics a balanced case-control study.

2 Conduct a single-marker regression model for GWAS and calculate a chi-square statistic for each marker.

3 Take $CS_i = \sum_j \chi_{1(j)}^2$ for the m simulation.

Repeat steps 1-3 for P rounds and calculate the sampling variance of $V_{CS} = var(CS)$. Then, the effective number of markers is consequently defined as

$$m_{e.1} = m \times \frac{2m}{V_{CS}} \qquad (12)$$

$m_{e.1}$ is interpreted as the number of independent chi-square tests, a key parameter that then simplified the simulation and analytical work for polygenic risk score (7).

4 General linkage disequilibrium and m_e

It is noticed that the global LD for all pair of markers

$$\ell_g = \frac{\sum_{l_1 l_2}^{m} r_{l_1 l_2}^2}{m^2} = \frac{1}{m_e} \tag{13}$$

Factors influencing LD can equivalently influence m_e. In Equation 6, let θ_{ij,l_1} be the relatedness score of a pair of individuals at the locus l_1^{th}. $var(\theta_{ij,l_1}) = \theta_{ii}\theta_{jj}r_{l_1 l_1}^2 + \theta_{ij}^2 r_{l_1 l_1}^2$, and $cov(\theta_{ij,l_1}, \theta_{ij,l_2}) = \theta_{ii}\theta_{jj}r_{l_1 l_2}^2 + \theta_{ij}^2 r_{l_1 l_2}^2$. The expected correlation of IBS between a pair of loci is $E(\gamma_{ij}) = \frac{(\theta_{ij}^2+1)r_{l_1 l_2}^2}{\sqrt{\theta_{ij}^2+1}\sqrt{\theta_{ij}^2+1}} = r_{l_1 l_2}^2$, which is not influenced by θ_{ij}. So, we only focus on population structure here.

The very basic equation for squared correlation r^2 can be written as (17)

$$r_{l_1 l_2}^2 = \frac{cov^2(x_{l1}, x_{l_2})}{var\,(x_{l_1})\,var(x_{l_2})} \tag{14}$$

In the absence of population structure, $r_{l_1 l_2}^2 = \frac{D_{l_1 l_2}^2}{4p_{l_1}q_{l_1}p_{l_2}q_{l_2}}$, the classic definition for LD between a pair of biallelic loci. However, in the presence of population structure, the analytical resolution for LD is unknown, but can be substantial given the subpopulations (22). Considering the l_1^{th} (l_2^{th}) locus, which is divided into $\mathcal{S}_1$ ($\mathcal{S}_2$) subpopulations, each of which is assumed to be under random mating, we could obtain the frequency distribution of the three genotypes (**Table 1**). For a pair of biallelic SNPs l_1 and l_2, the conditional probabilities of four haplotype phases ($a_{l_1}a_{l_2}$, $a_{l_1}A_{l_2}$, $A_{l_1}a_{l_2}$, and $A_{l_1}A_{l_2}$) are listed in **Table 2**, and we find the variance and covariance for the two SNPs. Ohta applied a similar treatment to the island model (23).

Genotype	$a_{l_1}a_{l_2}$	$A_{l_1}a_{l_2}$	$A_{l_1}A_{l_2}$	
Code	0	1	2	Marginal probability
Subpopulation 1	$q^2_{l_1(1)}$	$2p_{l_1(1)}q_{l_1(1)}$	$p^2_{l_1(1)}$	$w_1 = \frac{n_1}{n}$
Subpopulation 2	$q^2_{l_1(2)}$	$2p_{l_1(2)}q_{l_1(2)}$	$p^2_{l_1(2)}$	$w_2 = \frac{n_2}{n}$
$\vdots$	$\vdots$	$\vdots$	$\vdots$	$\vdots$
Subpopulation $\mathcal{S}_1$	$q^2_{l_1(\mathcal{S}_1)}$	$2p_{l_1(\mathcal{S}_1)}q_{l_1(\mathcal{S}_1)}$	$p^2_{l_1(\mathcal{S}_1)}$	$w_{\mathcal{S}_1} = \frac{n_{\mathcal{S}_1}}{n}$

Table 1: Frequency distribution of three possible genotypes at the l_1^{th} SNP in subpopulations 1, 2, till $\mathcal{S}_1$

		The l_2^{th} locus		
		a_{l_2}	A_{l_2}	Marginal probability
The l_1^{th} locus	a_{l_1}	$r_{l_1l_2} = q_{l_2} + \frac{D_{l_1l_2}}{q_{l_1}}$	$\bar{r}_{l_1l_2} = p_{l_2} - \frac{D_{l_1l_2}}{q_{l_1}}$	q_{l_1}
	A_{l_1}	$\bar{R}_{l_1l_2} = q_{l_2} - \frac{D_{l_1l_2}}{p_{l_1}}$	$R_{l_1l_2} = p_{l_2} + \frac{D_{l_1l_2}}{p_{l_1}}$	p_{l_1}
Marginal probability		q_{l_2}	p_{l_2}	

Table 2: Conditional probabilities of four haplotypes for SNPs l_1 and l_2. $r_{l_1l_2} = p(a_{l_1}|a_{l_2})$ and $R_{l_1l_2} = p(A_{l_1}|A_{l_2})$ are the conditional probabilities of the two coupling haplotypes. For the sake of convenience, we are going to use $\bar{r}_{l_1l_2} = 1 - r_{l_1l_2}$ and $\bar{R}_{l_1l_2} = 1 - R_{l_1l_2}$.

Haplotype	$a_{l_1}a_{a_2}$	$a_{l_1}A_{a_2}$	$A_{l_1}a_{a_2}$	$A_{l_1}A_{a_2}$
$a_{l_1}a_{l_2}$	$r^2_{l_1l_2}q^2_{l_1}$	$r_{l_1l_2}\bar{r}_{l_1l_2}q^2_{l_1}$	$r_{l_1l_2}\bar{r}_{l_1l_2}\ p_{l_1}q_{l_1}$	$r_{l_1l_2}R_{l_1l_2}p_{l_1}q_{l_1}$
$a_{l_1}A_{l_2}$	$r_{l_1l_2}\bar{r}_{l_1l_2}q^2_{l_1}$	$\bar{r}^2_{l_1l_2}q^2_{l_1}$	$\bar{r}_{l_1l_2}\bar{R}_{l_1l_2}p_{l_1}q_{l_1}$	$\bar{r}_{l_1l_2}R_{l_1l_2}p_{l_1}q_{l_1}$
$A_{l_1}a_{l_2}$	$\bar{R}_{l_1l_2}r_{l_1l_2}p_{l_1}q_{l_1}$	$\bar{r}_{l_1l_2}\bar{R}_{l_1l_2}\ p_{l_1}q_{l_1}$	$\bar{R}^2_{l_1l_2}p^2_{l_1}$	$\bar{R}_{l_1l_2}R_{l_1l_2}p^2_{l_1}$
$A_{l_1}A_{l_2}$	$R_{l_1l_2}r_{l_1l_2}p_{l_1}q_{l_1}$	$R_{l_1l_2}\bar{r}_{l_1l_2}p_{l_1}q_{l_1}$	$\bar{R}_{l_1l_2}R_{l_1l_2}p^2_{l_1}$	$R^2_{l_1l_2}p^2_{l_1}$

Table 3: Rearranged frequency distribution of the haplotype for a pair of biallelic SNPs l_1 and l_2

$$\begin{cases} \begin{cases} E(x_{l_1}) &=& \sum_{k=1}^{\mathcal{S}_1} w_k(2p_{l_{1(k)}}^2+2p_{l_{1(k)}}q_{l_{1(k)}})=2\bar{p}_{l_1} \\ E(x_{l_1}^2) &=& \sum_{k=1}^{\mathcal{S}_1} 2w_k p_{l_{1(k)}}q_{l_{1(k)}}+\sum_{k=1}^{\mathcal{S}_1} 4w_k p_{l_{1(k)}}^2 \\ var(x_{l_1}) &=& E(x_{l_1}^2)-E^2(x_{l_1})=2\sum_{i}^{\mathcal{S}_1} w_k[p_{l_{1(k)}}q_{l_{1(k)}}+2(p_{l_{1(k)}}^2-\bar{p}_{l_1}^2)] \end{cases} \\ \begin{cases} E(x_{l_2}) &=& \sum_{k=1}^{\mathcal{S}_2} w_k(2p_{l_{2(k)}}^2+2p_{l_{2(k)}}q_{l_{2(k)}})=2\bar{p}_{l_2} \\ E(x_{l_2}^2) &=& \sum_{k=1}^{\mathcal{S}_2} 2w_k p_{l_{2(k)}}q_{l_{2(k)}}+\sum_{k=1}^{\mathcal{S}_2} 4w_k p_{l_{2(k)}}^2 \\ var(x_{l_2}) &=& E(x_{l_2}^2)-E^2(x_{l_2})=2\sum_{k}^{\mathcal{S}_2} w_k[p_{l_{2(k)}}q_{l_{2(k)}}+2(p_{l_{2(k)}}^2-\bar{p}_{l_2}^2)] \end{cases} \\ \begin{cases} E(x_{l_1}x_{l_2}) &=& 2\sum_{k=1}^{\mathcal{S}_0} w_k D_{l_1 l_{2(k)}}+4\sum_{k=1}^{\mathcal{S}_0} w_k p_{l_{1(k)}}q_{l_{2(k)}} \\ cov(x_{l_1},x_{l_2}) &=& E(x_{l_1}x_{l_2})-E(x_{l_1})E(x_{l_2})=2\sum_{k=1}^{\mathcal{S}_0} w_k[D_{l_1 l_{2(k)}}+2(p_{l_{1(k)}}q_{l_{2(k)}}-\bar{p}_{l_1}\bar{p}_{l_2})] \end{cases} \end{cases} \tag{15}$$

These terms are sufficient to derive the general expression for LD. We plug the variance and covariance (**Equation 15**) into **Equation 14** and can further deduce a general expression for $\rho_{l_1 l_2}^2$. For simplicity, let $\mathcal{S}_0=\mathcal{S}_1=\mathcal{S}_2$, and we have

$$\tilde{r}_{l_1 l_2}^2=\frac{\{\sum_{k=1}^{\mathcal{S}_0} w_k[D_{l_1 l_{2(k)}}+2(p_{l_{1(k)}}p_{l_{2(k)}}-\bar{p}_{l_1}\bar{p}_{l_2})]\}^2}{\{\sum_{k=1}^{\mathcal{S}_0} w_k[p_{l_{1(k)}}q_{l_{1(k)}}+2(p_{l_{1(k)}}^2-\bar{p}_{l_1}^2)]\}\{\sum_{k=1}^{\mathcal{S}_0} w_k[p_{l_{2(k)}}q_{l_{2(k)}}+2(p_{l_{2(k)}}^2-\bar{p}_{l_2}^2)]\}} \tag{16}$$

When $\mathcal{S}_0=1$, $r_{l_1 l_2}^2=\frac{D_{l_1 l_2}^2}{p_{l_1}q_{l_1}p_{l_2}q_{l_2}}$, which is reduced to the conventional expression for squared LD metric. If we let $\mathcal{S}_0=2$, if $p_{l_1(1)}=1$, $p_{l_1(2)}=0$, and $p_{l_2(1)}=1$, $p_{l_2(2)}=0$, even $D_{l_1 l_2}=0$ it still produces $r_{l_1 l_2}^2=0.25$, an inflated LD driven by the population structure.

$$\begin{cases} m_e &=& \frac{m^2}{\sum_{l_1,l_2} r_{l_1 l_2}^2}=\frac{m^2}{m+\sum_{l_1\neq l_2} r_{l_1 l_2}^2} \\ \tilde{m}_e &=& \frac{m^2}{\sum_{l_1,l_2} \tilde{r}_{l_1 l_2}^2}=\frac{m^2}{m+\sum_{l_1\neq l_2} \tilde{r}_{l_1 l_2}^2} \end{cases} \tag{17}$$

When there is population structure, $m_e \geq \tilde{m}_e$. It is expected to observe strong shrinkage given increasing population structure.

References

[1] Stam, P. The distribution of the fraction of the genome identical by descent in finite random mating populations. *Genetical Research* **10**, 131–155 (1980).

[2] Visscher, P. M. *et al.* Assumption-free estimation of heritability from genome-wide identity-by-descent sharing between full siblings. *PLoS Genetics* **2**, e41 (2006). URL http://www.pubmedcentral.nih.gov/articlerender.fcgi?artid=1413498&tool=pmcentrez&rendertype=abstract.

[3] Hill, W. G. & Weir, B. S. Variation in actual relationship as a consequence of Mendelian sampling and linkage. *Genetics Research* **93**, 47–64 (2011). URL http://www.pubmedcentral.nih.gov/articlerender.fcgi?artid=3070763&tool=pmcentrez&rendertype=abstract.

[4] Goddard, M. Genomic selection: prediction of accuracy and maximisation of long term response. *Genetica* **136**, 245–257 (2009). URL http://www.ncbi.nlm.nih.gov/pubmed/18704696.

[5] Hayes, B. J., Visscher, P. M. & Goddard, M. E. Increased accuracy of artificial selection by using the realized relationship matrix. *Genetics Research* **91**, 47–60 (2009).

[6] Goddard, M. E., Hayes, B. J. & Meuwissen, T. H. Using the genomic relationship matrix to predict the accuracy of genomic selection. *Journal of Animal Breeding and Genetics* **128**, 409–421 (2011).

[7] Purcell, S. M. *et al.* Common polygenic variation contributes to risk of schizophrenia and bipolar disorder. *Nature* **460**, 748–752 (2009). URL http://www.ncbi.nlm.nih.gov/pubmed/19571811.

[8] Dudbridge, F. Power and predictive accuracy of polygenic risk scores. *PLoS Genetics* **9**, e1003348 (2013). URL http://www.pubmedcentral.nih.gov/articlerender.fcgi?artid=3605113&tool=pmcentrez&rendertype=abstract.

[9] Wang, X. *et al.* Polygenic risk prediction: why and when out-of-sample prediction R2 can exceed SNP-based heritability. *American Journal of Human Genetics* **110**, 1207–1215 (2023).

[10] Visscher, P. M. *et al.* Statistical power to detect genetic (co)variance of complex traits using SNP data in unrelated samples. *PLoS Genetics* **10**, e1004269 (2014). URL http://dx.plos.org/10.1371/journal.pgen.1004269.

[11] Chen, G.-B. Estimating heritability of complex traits from genome-wide association studies using IBS-based Haseman-Elston regression. *Frontiers in Genetics* **5**, 107 (2014). URL http://www.frontiersin.org/Statistical_Genetics_and_Methodology/10.3389/fgene.2014.00107/abstract.

[12] Visscher, P. M. & Goddard, M. E. A General Unified Framework To Assess the Sampling Variance of Heritability Estimates Using Pedigree or Marker-Based Relationships. *Genetics* **199**, 223–232 (2015). URL http://www.ncbi.nlm.nih.gov/pubmed/25361897.

[13] Zhou, X. A unified framework for variance component estimation with summary statistics in genome-wide association studies. *Annals of Applied Statistics* **11**, 2027–2051 (2017).

[14] Zhang, Q.-X. *et al.* Searching across-cohort relatives in 54, 092 GWAS samples via encrypted genotype regression. *PLoS Genetics* **20**, e1011037 (2024). URL http://dx.doi.org/10.1371/journal.pgen.1011037.

[15] Speed, D., Hemani, G., Johnson, M. R. & Balding, D. J. Improved Heritability Estimation from Genome-wide SNPs. *American Journal of Human Genetics* **91**, 1011–21 (2012). URL http://www.ncbi.nlm.nih.gov/pubmed/23217325.

[16] Qi, G.-A. *et al.* Analytical and computational solution for the estimation of SNP-heritability in biobank-scale and distributed datasets. *bioRxiv* 614017 (2024).

[17] Hill, W. G. & Robertson, A. The effect of linkage on limits to artificial selection. *Genetical Research* **8**, 269–294 (1966). URL http://www.journals.cambridge.org/abstract_S0016672300010156.

[18] Isserlis, L. On a formula for the product-moment coefficient of any order of a normal frequency distribution in any number of variables. *Biometrika* **12**, 134–139 (1918).

[19] Chang, C. C. *et al.* Second-generation PLINK: rising to the challenge of larger and richer datasets. *GigaScience* **4**, 7 (2015). URL http://gigascience.biomedcentral.com/articles/10.1186/s13742-015-0047-8. 1410.4803.

[20] Theodoris, C., Low, T. M., Pavlidis, P. & Alachiotis, N. quickLD: An efficient software for linkage disequilibrium analyses. *Molecular Ecology Resources* **21**, 2580–2587 (2021).

[21] Wu, Y. & Sankararaman, S. A scalable estimator of SNP heritability for biobank-scale data. *Bioinformatics* **34**, i187–i194 (2018).

[22] Nei, M. & Li, W.-h. LINKAGE DISEQUILIBRIUM IN SUBDIVIDED POPULATIONS. *Genetics* **75**, 213–219 (1973).

[23] Ohta, T. Linkage disequilibrium with the island model. *Genetics* **101**, 139–155 (1982).
