# Supplementary material for: Precise estimation of in-depth relatedness in biobank-scale datasets using deepKin
Source: Cell Rep Methods. 2025 May 27;5(6):101053. doi: 10.1016/j.crmeth.2025.101053 (PMC12272256; doi:10.1016/j.crmeth.2025.101053)
Supplement: Document S2. Article plus supplemental information [file mmc3.pdf]

Cell Reports Methods

Article

Precise estimation of in-depth relatedness in biobank-scale datasets using deepKin

Graphical abstract

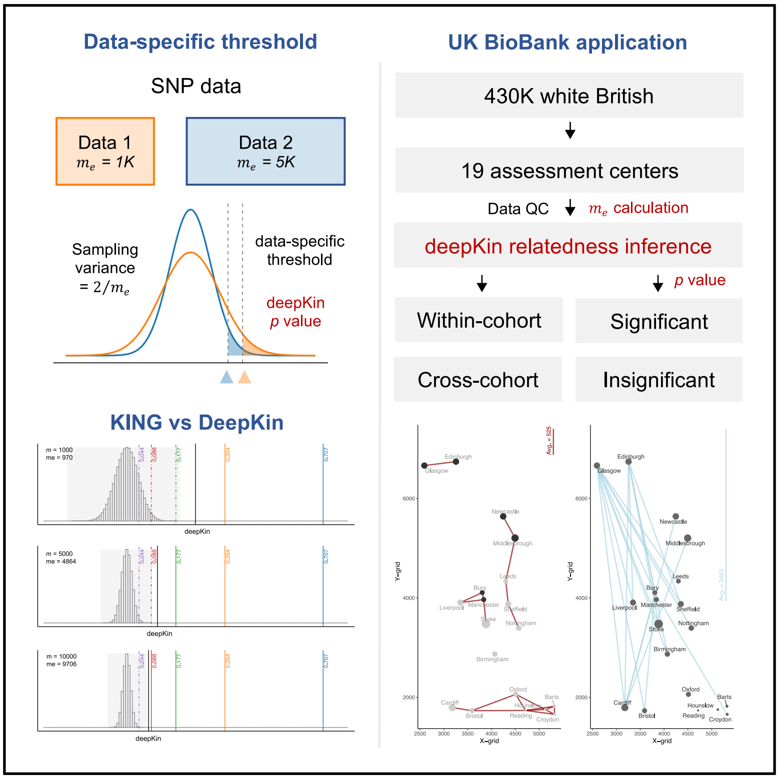

Authors

Qi-Xin Zhang (张齐心), Dovini Jayasinghe, Zhe Zhang (张哲), Sang Hong Lee, Hai-Ming Xu (徐海明), Guo-Bo Chen (陈国波)

Correspondence

zhangqx@zju.edu.cn (Q.-X.Z.), chenguobo@gmail.com (G.-B.C.)

In brief

Zhang et al. introduce deepKin, a moment-based method for estimating genetic relatedness with statistical significance. To incorporate data-specific sampling variance, deepKin emphasizes the impact of the effective number of markers. Applied to UKB, significant relatedness estimation reveals a “more diverse more related” pattern across 19 geographically distributed cohorts.

Highlights

- DeepKin enables inference of genetic relatedness with statistical significance
- Relationship inference depth is driven by the effective number of markers (m_e)
- Data-specific thresholds better control false positives vs. traditional fixed cutoffs
- Relatedness estimation reveals “geography-genotype” patterns across 19 UKB cohorts

Zhang et al., 2025, Cell Reports Methods 5, 101053
June 16, 2025 © 2025 The Authors. Published by Elsevier Inc.
https://doi.org/10.1016/j.crmeth.2025.101053

CellPress

Cell Reports Methods

CellPress
OPEN ACCESS

Article

Precise estimation of in-depth relatedness in biobank-scale datasets using deepKin

Qi-Xin Zhang (张齐心),[1,2,3,*] Dovini Jayasinghe,[3,4] Zhe Zhang (张哲),[5] Sang Hong Lee,[3,4] Hai-Ming Xu (徐海明),[1] and Guo-Bo Chen (陈国波)[2,6,7,*]

[1]Institute of Bioinformatics, Zhejiang University, Hangzhou, Zhejiang 310058, China
[2]Center for Laboratory Medicine, Department of Genetic and Genomic Medicine, and Clinical Research Institute, Zhejiang Provincial People's Hospital, People's Hospital of Hangzhou Medical College, Hangzhou, Zhejiang 310014, China
[3]Australian Centre for Precision Health and UniSA Allied Health and Human Performance, University of South Australia, Adelaide, SA 5000, Australia
[4]South Australian Health and Medical Research Institute (SAHMRI), University of South Australia, Adelaide, SA 5000, Australia
[5]Department of Animal Science, College of Animal Sciences, Zhejiang University, Hangzhou 310058, China
[6]Key Laboratory of Endocrine Gland Diseases of Zhejiang Province, Hangzhou, Zhejiang 310014, China
[7]Lead contact
*Correspondence: zhangqx@zju.edu.cn (Q.-X.Z.), chenguobo@gmail.com (G.-B.C.)
https://doi.org/10.1016/j.crmeth.2025.101053

MOTIVATION In the GWAS era, KING has become a widely used, rule-of-thumb method for inferring genetic relationships. However, a fundamental limitation remains overlooked: KING reports *k*-degree relatedness regardless of the number or characteristics of SNPs used. Intuitively, the precision of relatedness inference should depend on both the number of SNPs and their linkage disequilibrium structure. Moreover, the effects of sampling variance on inference have remained underexplored. These gaps motivate the need for a framework that accounts for marker-based sampling uncertainty to better enable data-specific inference of genetic relationships.

SUMMARY

Accurate relatedness estimation is essential in biobank-scale genetic studies. We present deepKin, a method-of-moments framework that accounts for sampling variance to enable statistical inference and classification of relatedness. Unlike traditional methods using fixed thresholds, deepKin computes data-specific significance thresholds, determines the minimum effective number of markers, and estimates the statistical power to detect distant relatives. Through simulations, we demonstrate that deepKin accurately infers both unrelated pairs and relatives by leveraging sampling variance. In the UK Biobank (UKB), analysis of the 3K Oxford subset showed that SNP sets with a larger effective number of markers provided greater power for detecting distant relatives. In the White British subset, deepKin identified over 212,000 significant relative pairs, categorized into six degrees, and revealed their geographic patterns across 19 UKB assessment centers through within-cohort and cross-cohort relatedness estimation. An R package (deepKin) is available at GitHub.

INTRODUCTION

Detecting relationships among samples is fundamental in genetics and epidemiological analysis, particularly in the context of genome-wide association studies (GWASs) and polygenic risk score.[1,2] Conventionally, relatedness has been estimated based on pedigrees, which represents the expected level of genetic similarity. However, with the abundance of genome-wide single nucleotide polymorphism (SNP) data, we can now measure realized relatedness that explicitly captures actual relationship.[3] However, SNP data themselves introduce complexity due to a variety of genotyping technologies, quality control (QC) procedures, and linkage disequilibrium (LD). Consequently, interpreting estimated relatedness based on genome-wide SNPs can be intricate.

There are various methods to estimate genome-wide relatedness, employing either maximum-likelihood approaches[4–6] or moment-based estimators.[7–10] Moment-based estimators are often preferred despite their lower precision because they are computationally efficient.[3] While few factors have been studied on influencing genome-wide relatedness estimations, Hill and Weir made an attempt to explore this within the framework of

© 2025 The Authors. Published by Elsevier Inc.
This is an open access article under the CC BY license (http://creativecommons.org/licenses/by/4.0/).

linkage analysis.[11] They have examined the variation for various pairwise relationships as a consequence of Mendelian sampling and linkage. In contrast, current practice on SNP-based measures is more embraced in the framework of population genome-wide association, while their variation has not been explored. The sampling variance of SNP-based measures varies depending on the LD of SNP data and the level of relatedness. For example, the sampling variance of estimate relatedness can be significantly larger when using more correlated SNPs (due to much tighter LD), impacting the statistical power to detect related pairs significantly deviated from unrelatedness. Although factors affecting the variation of method-of-moments relatedness estimators have not been fully explored, static cutoffs have been commonly adopted for inferring relatedness, such as kinship coefficients and identity by descent (IBD) coefficients.[8,12] Neglecting the sampling variance in inference may lead to false positives and misclassifications. Applying static cutoff thresholds without considering sampling variance, which is data specific, can generate spurious inferences of relatedness that may not be significantly different from unrelatedness, leading to false positives.[12]

In this study, we develop a moment-based framework for genome-wide relatedness inference, which is called deepKin. Distinguishing itself significantly from previous moment estimators, such as KING,[8] deepKin offers advanced capability in relatedness inference, supported by the following statistical features. One remarkable feature is its ability to evaluate and provide the sampling variance of the estimated relatedness. By leveraging its asymptotic distribution, deepKin facilitates the computation of p values for each relatedness pair, enabling the assessment of significant deviations from unrelatedness. Furthermore, key principles are emerging when performing relatedness estimation and relatedness inference. (1) deepKin determines the critical value that separates significant relatedness estimation from insignificant ones based on sampling variance, which we refer to as deepest significant relatedness; (2) it identifies the minimum effective number of markers required for detecting the target degree of relatives to be significantly different from unrelated pairs; and (3) given the target degree of relatedness, it provides the amount of statistical power to be improved or compromised. We verified the performance of deepKin through simulations and demonstrated its effectiveness using the UK Biobank dataset.[13] We also implemented deepKin estimation and the new inference framework in an R package named "deepKin," which is available in the GitHub repository (https://github.com/qixininin/deepKin).

RESULTS

Simulation results

We first validated the variance derived from deepKin in two models, the "single-locus model" and the "multiple-loci model." By simulating paired individuals with relatedness up to the third degree and calculating their relatedness estimation using KING-homo and deepKin, we presented their observed variances, together with the expected variances derived under the single-locus model (Figures 1A–1D). The observed variances of KING-homo and deepKin did not differ significantly from the expected one at all degrees and all minor allele frequency (MAF) scenarios. When a more generalized context was introduced, where MAFs were randomly assigned and different conditions of LD were considered, the observed variances resembled the expected variances derived under the multiple-loci model at all degrees of relatedness and fluctuated at different MAF scenarios (Figures 1E–1L). The two observed variances of KING-homo and deepKin showed no significant difference when the lower boundary of MAF was above 0.1. It is worth noting that the inclusion of low-frequency variants (MAF < 0.05) introduced a significant increase of sampling variance. With the choice of common variants, the expected variance is often conservative compared to the observed one.

We then performed a simulation to intuitively show the difference in the inference of relatedness between KING and deepKin (Figure 2). Only unrelated individuals were simulated. deepKin took into account the distribution of the null hypothesis, which was related to the effective number of markers of the data, and provided dynamic cutoffs for relatedness inference where all unrelated pairs were inferred as "insignificant" in all scenarios. The quantile plots for the p values of deepKin showed that the observed distribution matched our expectation. However, fixed thresholds of KING could lead to false positives either because there was limited number of markers or the target degree was too distant; for instance, the lower boundary for third degree (0.088) was within the distribution of unrelated individuals when m = 1,000 and 5,000, and the lower boundary for fourth degree (0.044) was within the distribution of unrelated individuals when m = 1,000, 5,000, and 10,000.

We also demonstrated the distribution of p values for different degrees of relatives (identical and first, second, third, and fourth degrees) in simulation (Figure 3). The closer the relatives, the more significant were their p values. It turned out that, when the actual size of m_e met the requirement of that of the target degree at the experiment-wise type I error rate of 0.05 and type II error rate of 0.1 based on guideline I, all target relatives were clearly separated from unrelated pairs based on the significant thresholds.

UK Biobank Oxford demo

We first confirmed the consistence of the genetic relationship matrix (GRM)-based and randomization-based estimators for m_e (Figure S1A). As the number of iterations (B) increases, the randomization-based estimation of m_e approaches the GRM-based estimation. We then described how m_e was fluctuated with different choices of markers in the Oxford demonstration (Figure S1B). A total of 13 conditions were made up of two procedures: random selection or LD pruning. By randomly selecting variants along the genome, m_e increased sharply at first and then slowly, eventually approximating that of the overall quality-controlled variants. For example, the number of markers and the effective number of markers were m = 298,211 and m_e = 7,634 for all SNPs. However, when the threshold of LD pruning was $r^2 < 0.4$, the number of markers and the effective number of markers were m = 91,670 and m_e = 41,000. Next, two regions with extreme LD conditions were considered to verify the value of m_e. Region I was recognized as the high LD region around the centromere of chromosome 11 (46.06–59.41 Mb), while

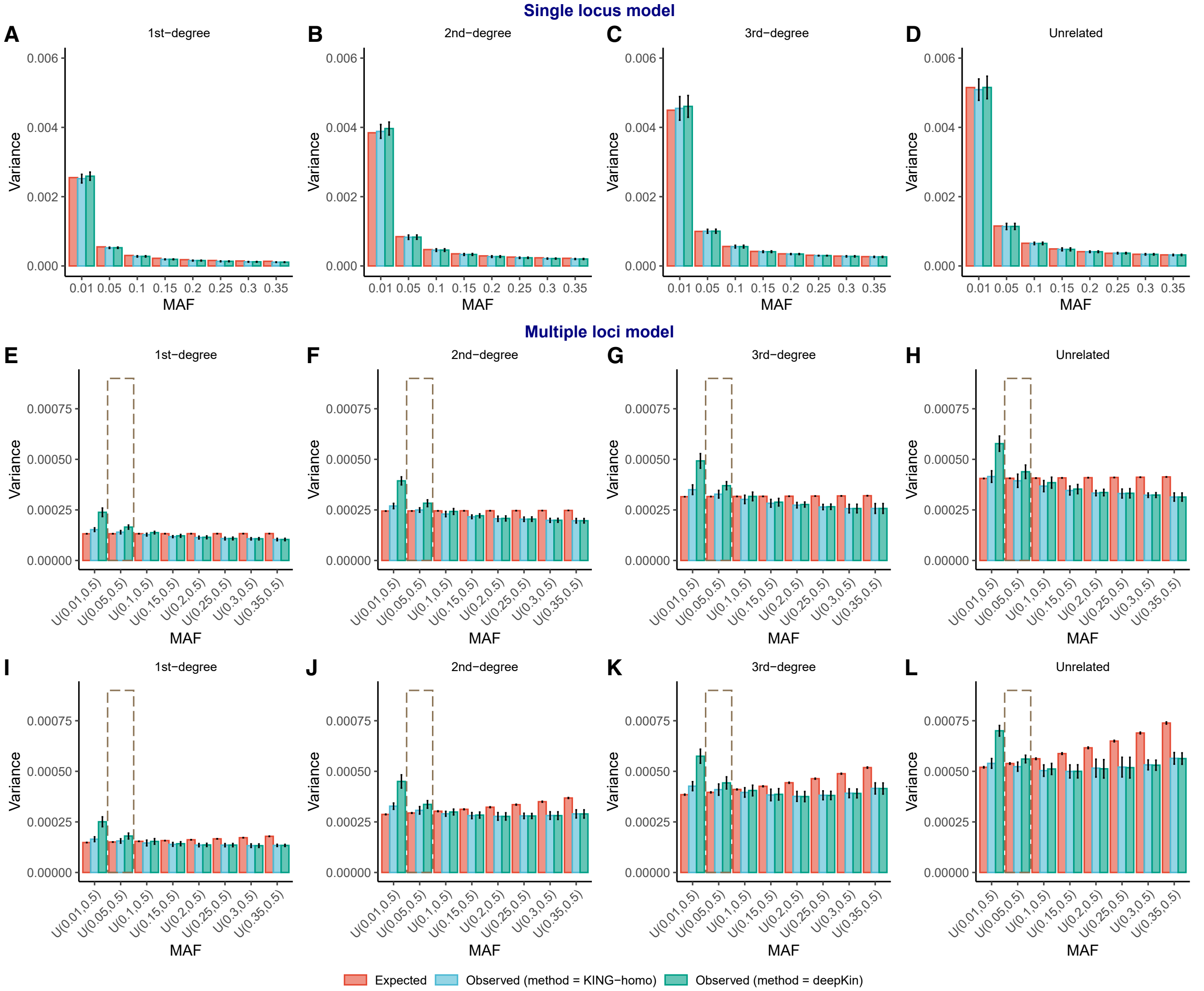

Figure 1. The expected and observed variances under "single-locus model" and "multiple-loci model" of different MAF scenarios in simulation

(A–D) Single-locus model: 5,000 independent markers of the same MAF (0.01, 0.05, 0.10, 0.15, 0.20, 0.25, 0.30, and 0.35) are simulated.

(E–L) Multiple-loci model: 5,000 dependent markers of different MAFs that are sampled from different uniform distributions are simulated, which are $U(0.01,0.5)$, $U(0.05,0.5)$, $U(0.10,0.5)$, $U(0.15, 0.5)$, $U(0.20,0.5)$, $U(0.25,0.5)$, $U(0.30,0.5)$, and $U(0.35,0.5)$. D' (Lewontin's measure) is used to depict the LD and is sampled from a uniform distribution $U(0.1, 0.2)$ and $U(0.5, 0.8)$ to represent low LD (E–H) and high LD (I–L). A total of 2,000 first-degree, second-degree, third-degree, and unrelated pairs are simulated and evaluated. All scenarios are performed in 10 repeats. 95% confidence intervals are given as error bars. The dashed box indicates the MAF threshold considered in following real-data analyses.

region II represented a generic region of low LD on chromosome 1 (0.75–33.45 Mb). The m_e values were extremely different for two regions with the same number of SNPs: $m_e = 490.1$ for region II but $m_e = 6.4$ for region I. Consequently, their expected deepKin variances were different. The expected variance of relatedness based on region I was too large to infer any conventional relationship, but based on region II, the deepKin threshold was able to distinguish certain relatedness (Figures S1C–S1F).

Based on these four sets of SNPs, we demonstrated the role of m_e in affecting the sampling variance of relatedness estimation and subsequently relatedness inference using 3,000 Oxford samples of White British ancestry. deepKin took m_e into consideration and calculated the deepest significant relatedness supported by each SNP set (Table 1). The degrees of deepest significant relatedness supported by SNP sets 1–3 were closer than the fourth degree, while SNP set 4, which had the largest size of m_e, harbored the deepest significant degree up to 4.409. Therefore, SNP set 4 discovered a total number of 57 significant relatedness, while SNP set 1–3 discovered 37, 32, and 38 significant relatedness, respectively (Table S1). We showed the expected power and the classification p values using these four sets of SNPs (Figures 4A and 4B). SNP set 4, which had the largest m_e and

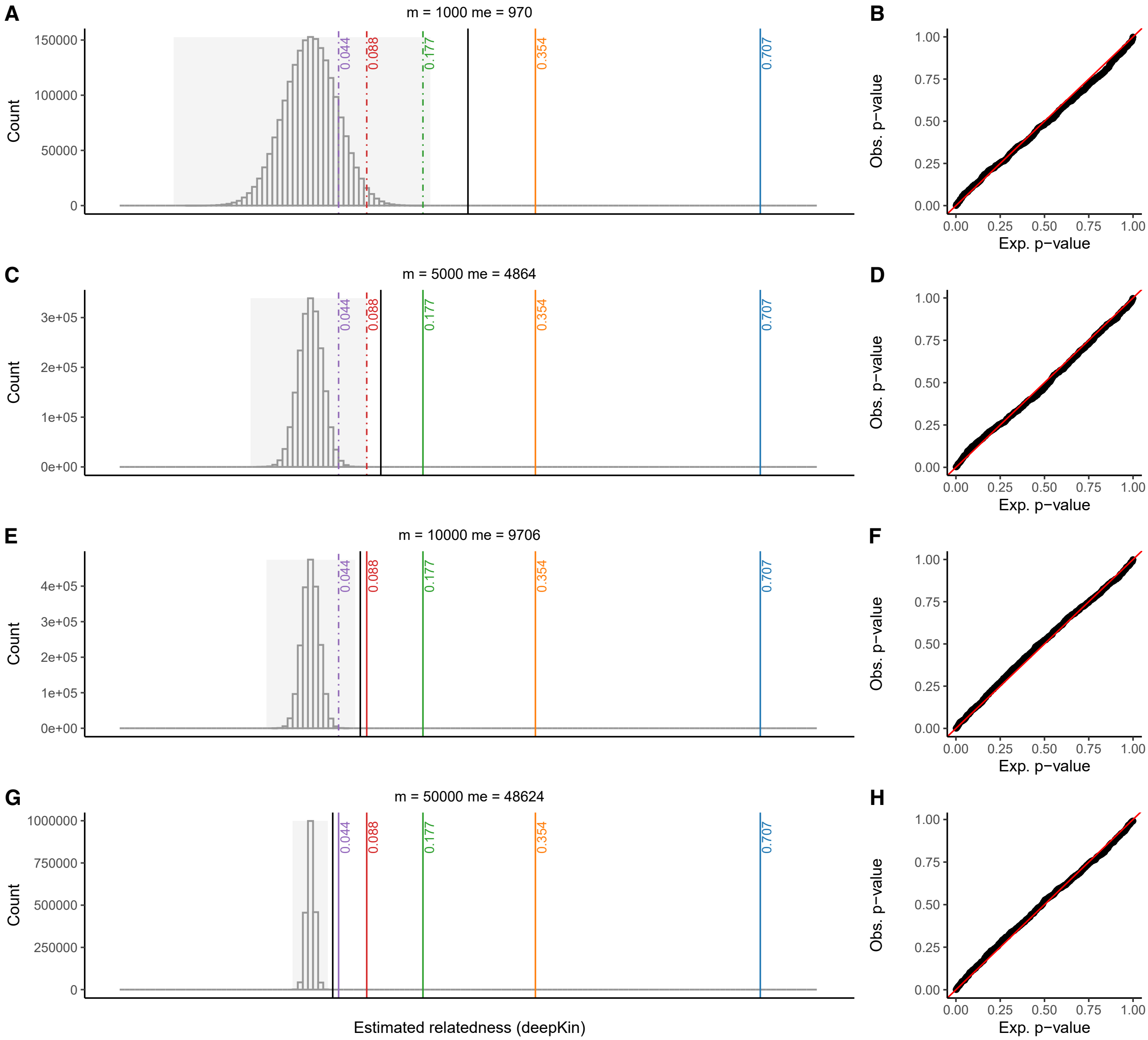

Figure 2. Different ways of relatedness inference between KING and deepKin

(A, C, E, and G) Histograms of relatedness score estimations by deepKin on unrelated individual pairs using different numbers of markers (m = 1,000, 5,000, 10,000, and 50,000). Only unrelated individuals (n = 2,000) are simulated. The line in black indicates the deepest significant relatedness calculated at the significant level of $\alpha = 0.05/1{,}999{,}000$. Lines in color indicate KING's lower boundaries of 0.707 (blue), 0.354 (yellow), 0.177 (green), 0.088 (red), and 0.044 (purple) for zero, first, second, third, and fourth degree of relatedness. Lower boundaries that are smaller than deepest relatedness supported by deepKin are plotted as dashed lines. The gray background indicates the observed relatedness range.

(B, D, F, and H) The quantile plots for deepKin p values. The p values on the x axis are sampled from the uniform distribution $U(0,1)$.

the smallest expected sampling variance, offered the most powerful inference for distant relatives and was the most reliable one in relatedness classification. Taking SNP sets 2 and 4 as examples, deepKin estimations were very consistent to the "KING-robust" estimations at positive values (Figures 4C and 4D). No additional relatives were found for deepKin under two different SNP sets. However, casual usage of KING's relative inference cutoffs, which were constant values regardless of the changing of SNP sets, might lead to substantial differences (Table 1).

To further investigate relatedness classification, the numbers of pairs assigned to each degree of relationship based on deepKin and KING were compared (Table 1). The numbers of relative pairs were quite consistent among the four SNP sets for identical and first-degree pairs (0 and 17, respectively) using deepKin or KING. The numbers of relative pairs were also quite consistent for second and third degree, 4 or 5 for second degree, and 8–11 for third degree. However, for fourth-degree relatives, the number of relative pairs discovered by KING inflated and

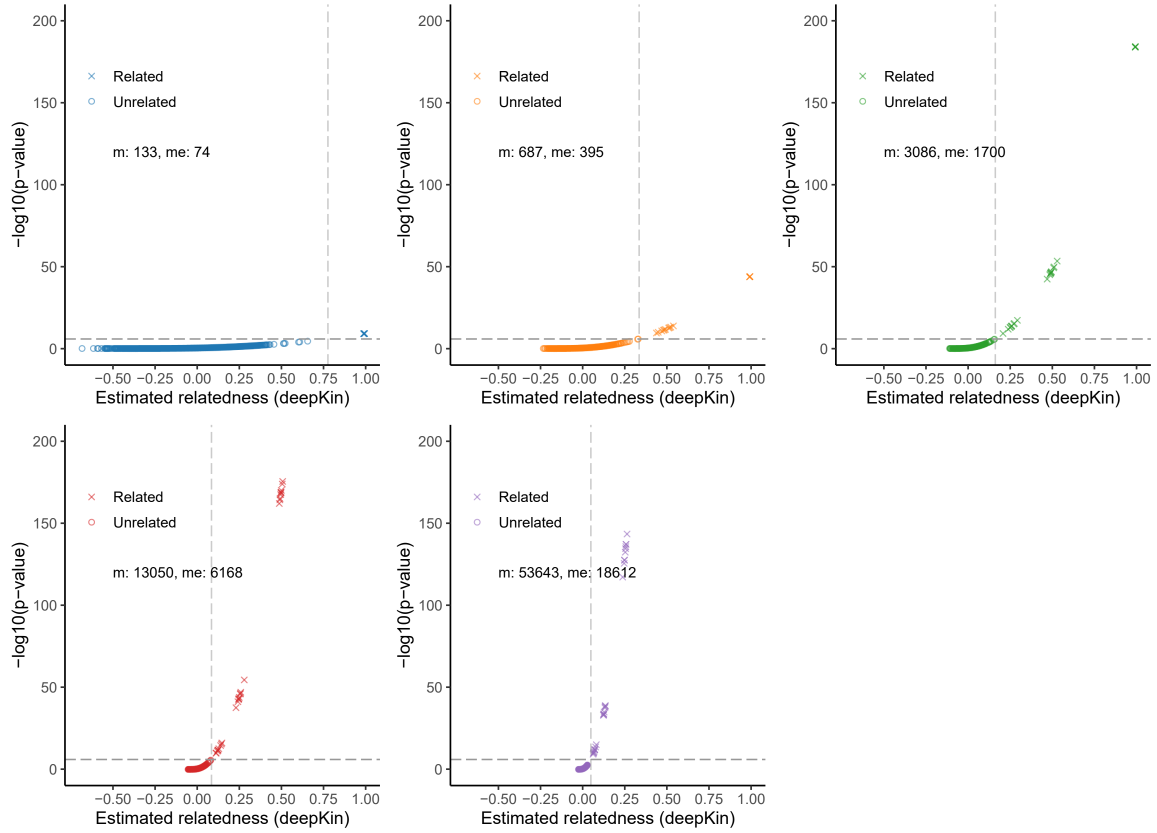

Figure 3. The distribution of *p* values for different degrees of relatives in simulation up to fourth degree
The plot shows the estimated relatedness scores and the corresponding *p* values for unrelated (circle) and related (cross) pairs. The horizontal line indicates the significant level of $\alpha = 0.05/40{,}000$. The vertical dashed line indicates the corresponding deepest significant relatedness based on Equation 5.

fluctuated dramatically among the four SNP sets, which were 2,769, 20,090, and 7,521 using SNP sets 1–3 and 209 using SNP set 4. As deepKin only performed classification on those significant signals, no abnormal inflation of distant relatives should be observed. Simple inference based on static criteria without considering the sampling variance could introduce unexpected false positives, at least for distant relatives who might be slightly related but were not fully supported by the data. The calculation of the deepest significant relatedness by deepKin offered safe examinations for each dataset and supported further relatedness classification.

As GRM is often used for sample QC in GWASs, we also investigated the number of pairs that exceeded the normal cutoffs for GRM (Table S2). Using different SNP sets, the numbers of excluded relative pairs ranged from 60 to 2,381 and from 655 to 72,032 using GRM's fixed cutoffs $\theta > 0.05$ and $\theta > 0.025$, respectively. These results provided a comprehensive understanding of how deepKin interacted with both the data itself and user-specified QCs.

As low-frequency variants might breach the asymptotic sampling variance for deepKin (Equation 4), we examined the sampling variance of deepKin estimations in the Oxford demo when using different MAF thresholds (0.01, 0.05, 0.10, 0.15, 0.20, 0.25, 0.30, and 0.35) (Figure S2). m was the number of variants that remained after applying MAF thresholds, and m_e was the effective number of markers calculated from the randomization-based method. There was no clear gap between the observed histogram and the asymptotic normal distribution with $\sigma^2 = 2/m_e$ when MAF thresholds were above 0.05. We strongly suggest that low-frequency variants should be removed during QC before applying deepKin.

UKB White British ancestry subset

We then applied deepKin to the full subset of UK Biobank (UKB) participants of White British ancestry (n = 427,287) from 19 assessment centers, excluding Stockport, Wrexham, and Swansea due to small sample sizes (<2,000). After basic QC and LD pruning, the m_e of the 72,016 markers was 56,945. We used deepKin to estimate nearly $\mathcal{N} \approx 9.13 \times 10^{10}$ pairs, which took approximate 193 h of wall time in total with 48 threads of two Intel Xeon CPU E5-2690 v3 at 2.60 GHz. A total of 232,552 (54.4%) UKB participants of White British ancestry were inferred to be related to at least one other person in the subset at the significant level of $0.05/\mathcal{N}$ and formed a total of 212,120 statistically significant related pairs (Figure 5A). Based on Equation 5, this SNP set held the deepest significant degree of $\delta = 4.567$ (95% confidence interval: 4.565–4.569). These 212,120 significantly relatedness estimations were classified into six degrees (Table S3): 162 pairs of identical

Table 1. QC details for four SNP sets and the numbers of pairs assigned to each degree of relationship based on the deepKin and KING framework in the Oxford demonstration

							Number of pairs	
SNP set	QC details	m	m_e	δ	θ^{δ}	Degree	deepKin	KING-robust
Set 1	MAF > 0.05	693,666	12,610	3.827	0.070	0	0	0
						1	17	17
						2	4	5
						3	10	8
						4	6	2,769
						5	insignificant	167,368
Set 2	MAF > 0.2	298,221	7,634	3.465	0.091	0	0	0
						1	17	17
						2	5	5
						3	9	11
						4	1	20,090
						5	insignificant	324,400
Set 3	MAF > 0.2, eigenGWAS p > 0.05	237,642	10,424	3.690	0.077	0	0	0
						1	17	17
						2	5	5
						3	9	10
						4	7	7,521
						5	insignificant	234,299
Set 4	MAF > 0.2; LD pruning (50 5 0.1)	36,425	28,244	4.409	0.047	0	0	0
						1	17	17
						2	4	5
						3	11	10
						4	25	209
						5	insignificant	44,270

3,000 individuals and a total number of ~4.5 million comparisons are considered (see also Table S1). m_e is estimated by randomization-based estimation. δ is the deepest significant degree the data would support to detect from unrelated individuals. θ^{δ} is the deepest significant relatedness score corresponding to δ, $\theta^{\delta} = (1/2)^{\delta}$.

pairs/monozygotic twins, 25,699 pairs of first-degree relatives, 9,455 pairs of second-degree relatives, and 53,221 pairs of third-degree relatives. Dividing by the total number of comparisons, $\mathcal{N}$, these forms were equivalent, but different numbers, to the relative components as reported in the original UKB report.[13] Besides, a total of 129,930 (30.4%) participants of White British ancestry were related up to at least third degree, where this ratio for all participants was 30.3% in Bycroft et al.[13] Moreover, since the deepest significant degree was deeper than third degree, we also reported 91,977 and 31,606 pairs of fourth- and fifth-degree relatives. Pairs of related individuals within the UKB White British subset formed networks of related individuals. While in most cases these were networks of size two or three, there were also many groups of size four or even larger in the subset (Figure 5B). If we only considered related individuals up to third degree, then the largest group size was reduced to 10 (data not shown).

We also analyzed the cross-cohort relatedness by assigning participants into these 19 cohorts based on their assessment centers. As anticipated, we discovered varying numbers of significant cross-cohort relatives with a notable pattern: the closer the geographical proximity, the more significant relatives identified (Figures 5C and 5D). We defined the proportion of cross-cohort significant relatives (P) as the ratio between the number of significant cross-cohort relatives (N_{sig}) and the total number of comparisons between the two cohorts (N). We observed several cohort pairs with a high proportion of cross-cohort significant relatives, including Manchester and Bury, Glasgow and Edinburgh, and Newcastle and Middlesbrough. The relative proportions of these three pairs were significantly higher than the other top 20 most related pairs. Conversely, certain cohort pairs, such as Glasgow and Cardiff, exhibited a low proportion of cross-cohort significant relatives, likely due to their considerable geographical distance. Specifically, the average grid distances for the top 20 and bottom 20 pairs, which had the highest and lowest P values, were 525 and 3,463, respectively (Figure 5C; Figure S3).

In each cohort, we considered its within-cohort averaging relatedness score ($\overline{\Theta}$) and significant relatedness score ($\overline{\Theta}_{sig}$), and we observed another notable pattern: the more diverse the population, the closer the relatives are within each cohort (Table 2; Figure 5E). Notably, Glasgow ($\overline{\Theta} = 0.00968$) and Edinburgh ($\overline{\Theta} = 0.00688$), both located in Scotland, exhibited higher levels of relatedness between individuals compared to other cohorts. On the

CellPress
OPEN ACCESS

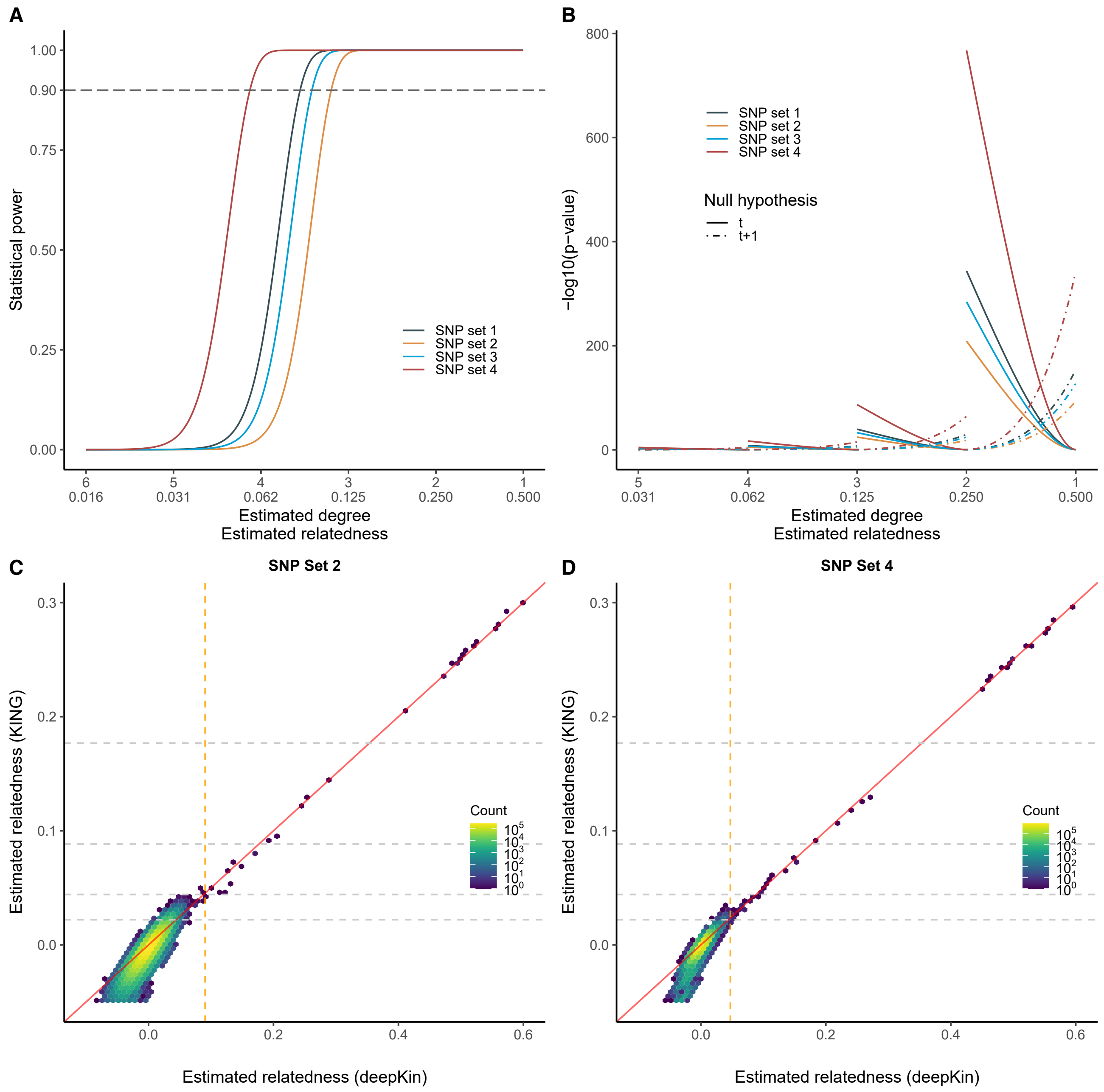

Figure 4. Oxford demonstration

(A) The expected power of detecting different relatedness using four SNP sets. The dashed gray line indicates 90% power. Supposed are a type I error rate of $\alpha = 0.05/N$ and type II error rate of $\beta = 0.1$, where $N = 4{,}498{,}500$.

(B) The expected p values for relatedness classification using four SNP sets. p values under two null hypotheses on adjacent t (solid curves) and $t+1$ (dashed curves) degrees are plotted.

(C and D) Scatterplots of the estimated relatedness by KING-robust and deepKin for all individual pairs in Oxford 3K demo using SNP set 2 (C) and SNP set 4 (D). Colors indicate the number of pairs that fall within the range of each hexagonal bin. Dashed gray lines on the y axis indicate KING's cutoffs, while the dashed orange line indicates deepKin's critical value at a significant level of $\alpha = 0.05/N$.

other hand, Barts displayed the lowest averaging relatedness ($\overline{\Theta} = -0.00160$), suggesting potential population diversity and even a slightly distinction in population structure (Figure S4). Intriguingly, Figure 5E reveals a sensible trend: a negative correlation (-0.809) between $\overline{\Theta}$ and $\overline{\Theta}_{sig}$. In particular, Barts, with the lowest $\overline{\Theta}$, showed the highest $\overline{\Theta}_{sig}$ of 0.262, while Glasgow, with the highest $\overline{\Theta}$, had the lowest $\overline{\Theta}_{sig}$ of 0.171. This trend remained significant even after adjusting for sample size or the number of significant

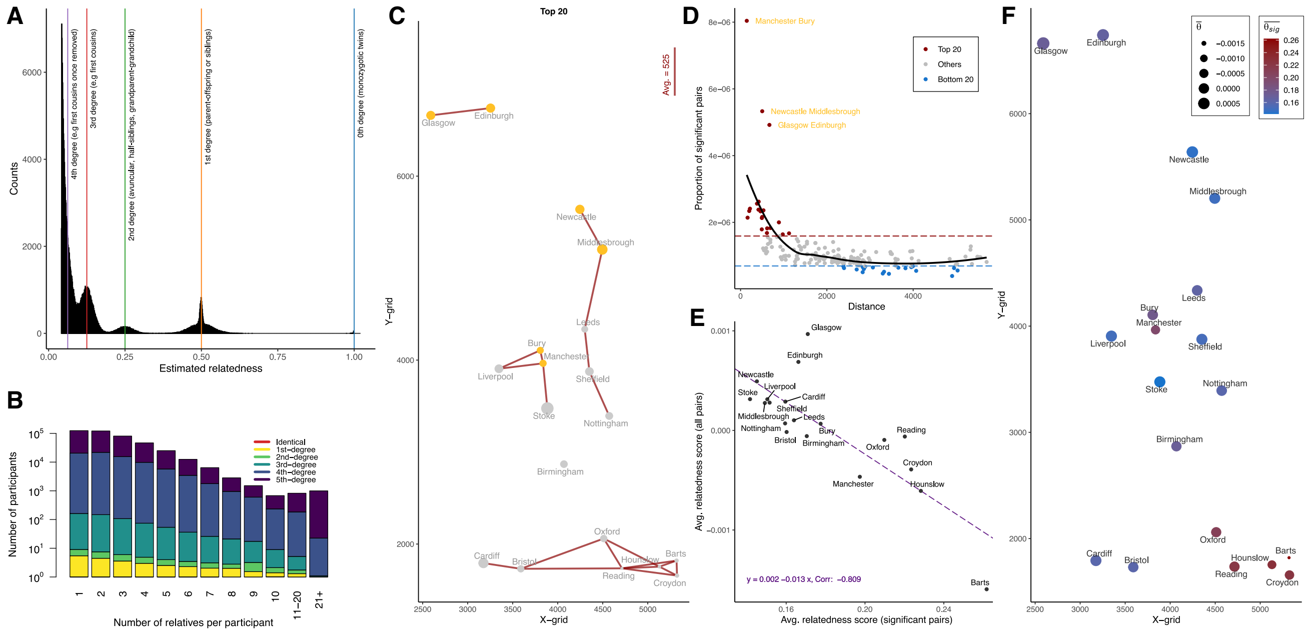

Figure 5. Relatedness in the UKB White British ancestry subset (n = 427,287)

(A) The histogram shows the distribution of relatedness estimation for 212,120 pairs of significant relatedness estimations inferred by deepKin. Colored lines indicate the expectation value of 1.000 (blue), 0.500 (yellow), 0.250 (green), 0.125 (red), and 0.0625 (purple) for zero-, first-, second-, third-, and fourth-degree relationships.

(B) Distribution of the number of related pairs that participants have in the UKB White British ancestry subset. The height of each bar shows the count of participants (log10 scale) with the stated number of relatives. The colors indicate the proportions of each degree of relatedness within a bar.

(C) Grid coordinates of 19 assessment centers in UKB and the top 20 pairwise proportion of cross-cohort significant relatives. The averaging distance is calculated from the average straight-line distance of the top 20 pairs of cohorts in the plot. The size of the dot indicates the size of the proportion of within-cohort significant relatives. Three special cohort pairs are shown in gold.

(D) The relationship between the proportion of cross-cohort significant relatives and pairwise distance. Three special cohort pairs are shown in gold.

(E) Regression plot of within-cohort averaging significant relatedness scores (x axis) and overall relatedness scores (y axis) for 19 assessment centers.

(F) Grid coordinates of 19 assessment centers in the UKB and within-cohort averaging significant relatedness scores (color scale) and averaging overall relatedness scores (size scale).

pairs within each cohort. These findings robustly indicated an increase in relatedness moving northward through London to Edinburgh or, in reverse, a potential increase in diversity from north to south across these 19 UKB cohorts.

DISCUSSION

Between KING and deepKin, the major difference is that deepKin presents missing elements of KING, such as sampling variance, and, consequently, sophisticated statistical inference can be conducted. It is known that the resolution of the relationship inference depends on the sampling variance of the estimator. As demonstrated previously for the IBD sharing inference, the sampling variance has been carefully calculated for relatives as defined in a traditional pedigree.[11] However, this general principle has not been fully established in the current framework of genome-wide relatedness measures using SNP data, even though KING has been proposed more than a decade ago and widely implemented in KING software and other popular GWAS tools, such as PLINK.[14] In this study, we present a moment-based estimator, deepKin, whose sampling variance has been analyzed to integrate the characteristics of data, such as MAF and LD, and eventually led to a practically useful asymptotic sampling variance. The availability of the sampling variance brings out a rigorous inference framework for moment-based relatedness estimators. Therefore, deepKin can assess p values for relatedness estimation, give statistical inference accordingly, divide them as “unrelated” and “related,” and refine classification of each pair into relatives of varying degrees.

Throughout the work, the effective number of markers plays a pivotal role in uncovering the sampling variance of the deepKin estimator under the multiple-loci model. The parameter m_e is a generic population parameter that characterizes the global LD of GWAS markers.[15] Compared to previous studies on the variation of IBD sharing, the counterpart parameter of m_e in linkage-style analysis is the genetic length of the genome measured in morgans.[11] Our deepKin guidelines may provide a general solution for choosing optimized SNPs for powerfully searching relatedness, since a larger m_e is more powerful for detecting deeper relatedness. It can be applied in more specific scenarios, such as designing optimized SNP chips for relatedness assessment in forensic applications.

SNP-based measures of genome similarity are highly sensitive to the MAFs. MAFs are influenced by factors such as the choice

Table 2. Summary for within-cohort relatedness for 19 UKB assessment centers

Cohort	X grid	Y grid	Sample size (n)	Total pairs (N)	Significant pairs (N_{sig})	$P\left(\frac{N_{sig}}{N}\right)$	$\overline{\Theta}$	$\overline{\Theta}_{sig}$
**Glasgow**	2,590	6,661	16,312	133,032,516	3,623	2.72E−05	**9.68E−04**	**0.171**
Edinburgh	3,254	6,740	15,442	119,219,961	3,364	2.82E−05	6.88E−04	0.166
Newcastle	4,246	5,639	34,683	601,437,903	17,340	2.88E−05	4.93E−04	0.145
Stoke	3,885	3,475	18,232	166,193,796	9,697	5.83E−05	3.14E−04	0.142
Liverpool	3,347	3,906	29,662	439,902,291	11,137	2.53E−05	3.13E−04	0.150
Cardiff	3,175	1,793	16,255	132,104,385	4,790	3.63E−05	2.89E−04	0.160
Sheffield	4,352	3,876	27,772	385,628,106	9,487	2.46E−05	2.79E−04	0.152
Middlesbrough	4,495	5,203	19,856	197,120,440	7,680	3.90E−05	2.75E−04	0.149
Leeds	4,300	4,336	39,707	788,303,071	11,362	1.44E−05	1.02E−04	0.164
Nottingham	4,572	3,393	30,801	474,335,400	9,197	1.94E−05	7.06E−05	0.159
Bury	3,806	4,106	25,421	323,100,910	4,959	1.53E−05	6.70E−05	0.177
Bristol	3,590	1,731	39,162	766,811,541	12,548	1.64E−05	−1.59E−05	0.160
Birmingham	4,069	2,869	19,596	191,991,810	3,511	1.83E−05	−5.73E−05	0.170
Reading	4,714	1,736	25,882	334,926,021	1,873	5.59E−06	−6.17E−05	0.220
Oxford	4,511	2,060	12,265	75,208,980	1,264	1.68E−05	−9.57E−05	0.210
Croydon	5,325	1,657	18,568	172,376,028	1,115	6.47E−06	−3.92E−04	0.223
Manchester	3,837	3,965	11,517	66,314,886	1,070	1.61E−05	−4.66E−04	0.197
Hounslow	5,130	1,754	18,453	170,247,378	986	5.79E−06	−6.07E−04	0.228
**Barts**	5,320	1,820	7,701	29,648,850	190	6.41E−06	**−1.60E−03**	**0.262**

N is the total number of pairs within each cohort, and $N = \frac{n(n-1)}{2}$. Stockport, Wrexham, and Swansea were excluded due to small sample sizes (<2,000). N_{sig} is the number of pairs whose p value is smaller than the cutoff of $0.05/\mathcal{N}$, and $\mathcal{N} \approx 9.13 \times 10^{10}$ for all pairs of comparisons. P is defined as the proportion of significant pairs within each cohort. $\overline{\Theta}$ is the averaging relatedness score of N pairs within each cohort. $\overline{\Theta}_{sig}$ is the averaging relatedness score of N_{sig} significant pairs within each cohort.

of SNP genotyping technology and QC procedures. Although the expected variance under the single-locus model showed high precision in simulation, it is not practical to assume the same MAF for all variants. At this moment, we extended our derivation to a multiple-loci model by assuming a normal distribution of the genotypes after locus-wise standardization, but this assumption may be violated when many low-frequency variants are included. We suggest removing low-frequency variants and possibly avoiding false positives. Our results demonstrated that the asymptotic variance based on the multiple-loci model performed well in both simulations and real dataset applications; future work should focus on extending the assumption of binomial distribution to multiple loci, which takes into consideration the distribution of both MAF and LD.

Although we have demonstrated, in principle, the theoretical merits and utility of deepKin, there are several questions that should be taken into account for its further development. When comparing deepKin with KING, our analysis primarily focused on KING-homo, which performs relatedness estimation and inference within a homogeneous population. deepKin prefers to employ the strategy of selecting non-ancestry informative markers (non-AIMs) to mitigate the potential influence of differentiated allele frequencies. The influence of population stratification on m_e and, thus, the sampling variance of relatedness estimation is still under investigation (Methods S1). One further investigation is on the union of unilineal relatives and bilineal relatives when detangling the variance of the estimator. In most of the application studies that are based on genome-wide similarity measures, first degree is usually where they stopped when distinguishing unilineal relatives (parent-offspring) and bilineal relatives (full siblings). Analogously, the method could likely be extended to biallelic sharing, commonly referred to as IBD2. Achieving the sampling variance of IBD2 would require further theoretical development and represents an intriguing direction for the next stage of our work. This study, by all means, will be helpful in the construction of a more systematic framework on performing statistical inference on genome-wide similarity measures.

Limitations of the study

deepKin currently assumes a relatively homogeneous population and addresses population structure by excluding AIMs, but residual stratification may still influence the effective number of markers and relatedness inference. Additionally, deepKin does not yet differentiate between unilineal and bilineal relationships, such as parent-offspring versus full siblings. These limitations highlight areas for future theoretical and methodological development.

RESOURCE AVAILABILITY

Lead contact

Requests for further information, resources, and data should be directed to and will be fulfilled by the lead contact, Guo-Bo Chen (chenguobo@gmail.com).

Materials availability

This study analyzed publicly available data and did not generate new reagents.

Data and code availability

- This study analyzed genotype and phenotype data provided by the UK Biobank, which can be accessed through procedures described on its webpage (https://www.ukbiobank.ac.uk/).
- The deepKin estimation and inference have been implemented in an R package, deepKin. DeepKin can be downloaded from https://github.com/qixininin/deepKin. An archival DOI is provided in the key resources table.
- Any additional information required to re-analyze the data reported in this paper is available from the lead contact upon request.

ACKNOWLEDGMENTS

We thank the participants of the UK Biobank for making this work possible (application ID 41376). This work was supported by National Natural Science Foundation of China (31771392 to G.-B.C. and 32102503 to Z.Z.). Q.-X.Z. thanks the China Scholarship Council program (project ID 202306320498) for support.

AUTHOR CONTRIBUTIONS

Conceptualization, G.-B.C.; data curation, Q.-X.Z.; formal analysis, Q.-X.Z.; funding acquisition, G.-B.C. and Z.Z.; investigation, Q.-X.Z. and G.-B.C.; methodology, Q.-X.Z., Z.Z., S.H.L., and G.-B.C.; project administration, G.-B.C.; resources, S.H.L., H.-M.X., and G.-B.C.; software, Q.-X.Z. and D.J.; supervision, H.-M.X. and G.-B.C.; writing – original draft, Q.-X.Z. and G.-B. C.; writing – review & editing, all authors.

DECLARATION OF INTERESTS

The authors declare no competing interests.

STAR★METHODS

Detailed methods are provided in the online version of this paper and include the following:

- KEY RESOURCES TABLE
- METHOD DETAILS
  - KING's estimator
  - deepKin estimator
  - Variance of deepKin under the single-locus model
  - Variance of deepKin under the multiple-loci model
  - Relatedness inference: Deepest significant estimation
  - Relatedness inference: Classification
  - Derived guidelines from deepKin
  - On the effective number of markers (m_e)
  - Simulation
  - UK Biobank application
- QUANTIFICATION AND STATISTICAL ANALYSIS

SUPPLEMENTAL INFORMATION

Supplemental information can be found online at https://doi.org/10.1016/j.crmeth.2025.101053.

Received: August 1, 2024
Revised: February 2, 2025
Accepted: April 29, 2025
Published: May 27, 2025

REFERENCES

1. Choi, S.W., Mak, T.S.H., and O'Reilly, P.F. (2020). Tutorial: a guide to performing polygenic risk score analyses. Nat. Protoc. *15*, 2759–2772. https://doi.org/10.1038/s41596-020-0353-1.
2. Uffelmann, E., Huang, Q.Q., Munung, N.S., de Vries, J., Okada, Y., Martin, A.R., Martin, H.C., Lappalainen, T., and Posthuma, D. (2021). Genome-wide association studies. Nat. Rev. Methods Primers *1*, 59. https://doi.org/10.1038/s43586-021-00056-9.
3. Speed, D., and Balding, D.J. (2015). Relatedness in the post-genomic era: Is it still useful? Nat. Rev. Genet. *16*, 33–44. https://doi.org/10.1038/nrg3821.
4. Thompson, E.A. (1975). The estimation of pairwise relationships. Ann. Hum. Genet. *39*, 173–188. https://doi.org/10.1111/j.1469-1809.1975.tb00120.x.
5. Milligan, B.G. (2003). Maximum-Likelihood Estimation of Relatedness. Genetics *163*, 1153–1167. https://doi.org/10.1093/genetics/163.3.1153.
6. Choi, Y., Wijsman, E.M., and Weir, B.S. (2009). Case-control association testing in the presence of unknown relationships. Genet. Epidemiol. *33*, 668–678. https://doi.org/10.1002/gepi.20418.
7. VanRaden, P.M. (2008). Efficient Methods to Compute Genomic Predictions. J. Dairy Sci. *91*, 4414–4423. https://doi.org/10.3168/jds.2007-0980.
8. Manichaikul, A., Mychaleckyj, J.C., Rich, S.S., Daly, K., Sale, M., and Chen, W.M. (2010). Robust relationship inference in genome-wide association studies. Bioinformatics *26*, 2867–2873. https://doi.org/10.1093/bioinformatics/btq559.
9. Yang, J., Benyamin, B., McEvoy, B.P., Gordon, S., Henders, A.K., Nyholt, D.R., Madden, P.A., Heath, A.C., Martin, N.G., Montgomery, G.W., et al. (2010). Common SNPs explain a large proportion of heritability for human height. Nat. Genet. *42*, 565–569. https://doi.org/10.1038/NG.608.
10. Conomos, M.P., Reiner, A.P., Weir, B.S., and Thornton, T.A. (2016). Model-free Estimation of Recent Genetic Relatedness. Am. J. Hum. Genet. *98*, 127–148. https://doi.org/10.1016/J.AJHG.2015.11.022.
11. Hill, W.G., and Weir, B.S. (2011). Variation in actual relationship as a consequence of Mendelian sampling and linkage. Genet. Res. *93*, 47–64. https://doi.org/10.1017/S0016672310000480.
12. Ramstetter, M.D., Dyer, T.D., Lehman, D.M., Curran, J.E., Duggirala, R., Blangero, J., Mezey, J.G., and Williams, A.L. (2017). Benchmarking relatedness inference methods with genome-wide data from thousands of relatives. Genetics *207*, 75–82. https://doi.org/10.1534/GENETICS.117.1122/-/DC1.
13. Bycroft, C., Freeman, C., Petkova, D., Band, G., Elliott, L.T., Sharp, K., Motyer, A., Vukcevic, D., Delaneau, O., O'Connell, J., et al. (2018). The UK Biobank resource with deep phenotyping and genomic data. Nature *562*, 203–209. https://doi.org/10.1038/s41586-018-0579-z.
14. Purcell, S., Neale, B., Todd-Brown, K., Thomas, L., Ferreira, M.A.R., Bender, D., Maller, J., Sklar, P., De Bakker, P.I.W., Daly, M.J., and Sham, P.C. (2007). PLINK: A Tool Set for Whole-Genome Association and Population-Based Linkage Analyses. Am. J. Hum. Genet. *81*, 559–575. https://doi.org/10.1086/519795.
15. Huang, X., Zhu, T.-N., Liu, Y.-C., Qi, G.A., Zhang, J.N., and Chen, G.B. (2023). Efficient estimation for large-scale linkage disequilibrium patterns of the human genome. Elife *12*, RP90636. https://doi.org/10.7554/eLife.90636.
16. Isserlis, L. (1918). On a Formula for the Product-Moment Coefficient of any Order of a Normal Frequency Distribution in any Number of Variables. Biometrika *12*, 134. https://doi.org/10.2307/2331932.
17. Almarri, M.A., Haber, M., Lootah, R.A., Hallast, P., Al Turki, S., Martin, H.C., Xue, Y., and Tyler-Smith, C. (2021). The genomic history of the Middle East. Cell *184*, 4612–4625.e14. https://doi.org/10.1016/j.cell.2021.07.013.
18. Goddard, M. (2009). Genomic selection: Prediction of accuracy and maximisation of long term response. Genetica *136*, 245–257. https://doi.org/10.1007/s10709-008-9308-0.

CellPress
OPEN ACCESS

19. Chen, G.B. (2014). Estimating heritability of complex traits from genome-wide association studies using IBS-based Haseman-Elston regression. Front. Genet. *5*, 107. https://doi.org/10.3389/FGENE.2014.00107/ABSTRACT.

20. Visscher, P.M., Hemani, G., Vinkhuyzen, A.A.E., Chen, G.B., Lee, S.H., Wray, N.R., Goddard, M.E., and Yang, J. (2014). Statistical Power to Detect Genetic (Co)Variance of Complex Traits Using SNP Data in Unrelated Samples. PLoS Genet. *10*, e1004269. https://doi.org/10.1371/journal.pgen.1004269.

21. Zhou, X. (2017). A unified framework for variance component estimation with summary statistics in genome-wide association studies. Ann. Appl. Stat. *11*, 2027–2051. https://doi.org/10.1214/17-AOAS1052.

22. Wu, Y., and Sankararaman, S. (2018). A scalable estimator of SNP heritability for biobank-scale data. Bioinformatics *34*, i187–i194. https://doi.org/10.1093/bioinformatics/bty253.

23. Girard, A. (1989). A fast 'Monte-Carlo cross-validation' procedure for large least squares problems with noisy data. Numer. Math. *56*, 1–23. https://doi.org/10.1007/BF01395775.

24. Hutchinson, M.F. (1990). A stochastic estimator of the trace of the influence matrix for laplacian smoothing splines. Commun. Stat. Simulat. Comput. *19*, 433–450. https://doi.org/10.1080/03610919008812866.

25. Chen, G.B., Lee, S.H., Zhu, Z.X., Benyamin, B., and Robinson, M.R. (2016). EigenGWAS: Finding loci under selection through genome-wide association studies of eigenvectors in structured populations. Heredity *117*, 51–61. https://doi.org/10.1038/hdy.2016.25.

STAR★METHODS

KEY RESOURCES TABLE

REAGENT or RESOURCE	SOURCE	IDENTIFIER
Deposited data		
UK Biobank	Bycroft et al.[13]	https://www.ukbiobank.ac.uk/
Software and algorithms		
deepKin	This paper	https://github.com/qixininin/deepKin<br>https://doi.org/10.5281/zenodo.15220287
KING	Manichaikul et al.[8]	https://www.kingrelatedness.com/
PLINK	Purcell et al.[14]	https://www.cog-genomics.org/plink/2.0/
X-LD	Huang et al.[15]	https://github.com/gc5k/gear2

METHOD DETAILS

One of the attempts of the study is to explore the sampling variance for moment-based genetic relatedness. We propose the deepKin estimator, which resembles the KING's original estimator and differs in the choice of genotype scaling factors. Two forms of genetic relationship matrix (GRM) can be found in Speed and Balding's review (see Equation 8; Equation 9 in their review)[3] or in VanRaden,[7] which are $g_{ij} = \frac{\sum_l^m (x_{il} - 2\hat{p}_l)(x_{jl} - 2\hat{p}_l)}{\sum_l^m 2\hat{p}_l(1-\hat{p}_l)}$ and $\tilde{g}_{ij} = \frac{1}{m}\sum_l^m \frac{x_{il} - 2\hat{p}_l}{\sqrt{2\hat{p}_l(1-\hat{p}_l)}} \frac{x_{jl} - 2\hat{p}_l}{\sqrt{2\hat{p}_l(1-\hat{p}_l)}} = \frac{1}{m}\tilde{x}_{il}\tilde{x}_{jl}$. Here, x_{il} and x_{jl} are the genotypic values (0, 1, or 2, according to the number of reference alleles) at l locus for individuals i and j, while $\hat{p}_l$ is the allele frequency at l-th locus and m is the total number of variants.

KING's estimator

The original kinship estimator of KING is (Equation 5 in their original publication[8])

$$\hat{\theta}_K = 2\hat{\phi}_{ij} = 1 - \frac{1}{2}\frac{\sum_l^m (x_{il} - x_{jl})^2}{\sum_l^m 2\hat{p}_l(1-\hat{p}_l)} \quad \text{(Equation 1)}$$

Here the definition of $\hat{\phi}_{ij}$ is the same as that in KING's paper but the expectation of KING's estimator is only half of the relatedness θ. Therefore, in the following text, we tend to use 2 times of the KING's estimator ($\hat{\theta}_K = 2\hat{\phi}_{ij}$) so as to make a clearer comparison. θ has the expected values of 1, 0.5, 0.25, and 0.125 for the zero- (monozygotic twin), first- (full sib, or parent-offspring), second- (half sib, or grandparent-grandchild), and third-degree (first cousin, or great grandparent-great grandchild) relatives, respectively. However, measured through genome-wide similarity, the realized value of θ could cover any value between 0 and 1. Note that, the sampling variance of θ_K has not been explored since it was proposed and the absence of sampling variance hinders the feasibility of conducting precise statistical tests in the statistical framework.

Degrees of relatedness with expected relatedness scores (θ) and kinship coefficients (ϕ)

		θ		ϕ	
Degree	Relationships	Expectation	Lower boundary	Expectation	Lower boundary
0	monozygotic twins	$\frac{1}{2^0} = 1$	0.707	$\frac{1}{2^1} = 0.5$	0.354
1	parent-offspring or siblings	$\frac{1}{2^1} = 0.5$	0.354	$\frac{1}{2^2} = 0.25$	0.177
2	avuncular, half-siblings, grandparent-grandchild	$\frac{1}{2^2} = 0.25$	0.177	$\frac{1}{2^3} = 0.125$	0.088
3	first cousins	$\frac{1}{2^3} = 0.125$	0.088	$\frac{1}{2^4} = 0.0625$	0.044
t	t-th degree relationships	$\frac{1}{2^t}$	$\frac{1}{2^{(2t+1)/2}}$	$\frac{1}{2^{t+1}}$	$\frac{1}{2^{(2t+3)/2}}$

The table includes example relationships for each degree and the corresponding lower boundaries from KING. The table does not include all possible relationship types for these degrees of relatedness. The table shows relatedness up to the t-th degree. Actually, in KING's original publication, they only considered relatedness up to third degree, and any relatedness score <0.088 is considered unrelated.

deepKin estimator

Inspired by the construction of two GRMs with different scaling factors and in contrast to Equation 1, the deepKin estimator ($\widehat{\theta}_D$) is constructed as,

$$\widehat{\theta}_D = 1 - \frac{1}{2}\frac{1}{m}\sum_{l}^{m}\left[\frac{x_{il} - 2\widehat{p}_l}{\sqrt{2\widehat{p}_l(1-\widehat{p}_l)}} - \frac{x_{jl} - 2\widehat{p}_l}{\sqrt{2\widehat{p}_l(1-\widehat{p}_l)}}\right]^2 = 1 - \frac{1}{2}\frac{1}{m}\sum_{l}^{m}(\tilde{x}_{il} - \tilde{x}_{jl})^2 \qquad \text{(Equation 2)}$$

After some rearrangement, Equation 2 can be decomposed into three parts, which are $\frac{1}{m}\sum_l^m (\tilde{x}_{il} - \tilde{x}_{jl})^2 = \frac{1}{m}\sum_l^m \tilde{x}_{il}^2 + \frac{1}{m}\sum_l^m \tilde{x}_{jl}^2 - \frac{2}{m}\sum_l^m \tilde{x}_{il}\tilde{x}_{jl} = \tilde{g}_{ii} + \tilde{g}_{jj} - 2\tilde{g}_{ij}$. Both $\tilde{g}_{ii}$, $\tilde{g}_{jj}$, and $\tilde{g}_{ij}$ are matrix elements of the GRM, which has the computational cost of $\mathcal{O}(n^2m)$. This decomposition gives the relationship between GRM and deepKin that $\widehat{\theta}_D$ for any pair of individuals can be quickly realized through addition of these three GRM elements. The computational cost of deepKin is consequently $\mathcal{O}(n^2m)$, which is identical to the cost of constructing GRM.

We first give the expectation and variance of deepKin under the assumption of a binomial distribution at one locus, namely the "single-locus model" (see section "Variance of deepKin under the single-locus model"). The expectation and variance of deepKin for a marker locus l ($\widehat{\theta}_{D_l}$) are

$$E\left(\widehat{\theta}_{D_l}\right) = \theta \text{ and } var\left(\widehat{\theta}_{D_l}\right) = \frac{1}{2}(1-\theta)^2 + \frac{1-\theta}{4\widehat{p}_l\widehat{q}_l} \qquad \text{(Equation 3)}$$

However, in practice we have many variants that are often in LD, therefore, we derive the expectation and variance of deepKin under the "multiple-loci model" (see section "Variance of deepKin under the multiple-loci model"). We assume that the aggregated standardized genotypes asymptotically follow the normal distribution and have the expectation of $E(\tilde{x}_{il}) = E(\tilde{x}_{jl}) = 0$ and variance of $var(\tilde{x}_{il}) = var(\tilde{x}_{jl}) = 1$, which meets the requirement of Isserlis's theorem.[16] After recursively applying Isserlis's theorem, we are able to derive the expectation and asymptotic variance of $\widehat{\theta}_D$, which are,

$$E(\widehat{\theta}_D) = \theta \text{ and } var(\widehat{\theta}_D) = \frac{2(1-\theta)^2}{m_e} \qquad \text{(Equation 4)}$$

The sampling variance of $\widehat{\theta}_D$ between two individuals is subject to the true relatedness (θ, lessened sampling variance for more related samples) and the global LD between variants ($m_e = m^2/\sum_{l_1,l_2}^m \rho_{l_1l_2}^2$ is the effective number of markers, in which $\rho_{l_1l_2}^2$ is squared correlation between loci l_1 and l_2; see more discussion on m_e below). $var(\widehat{\theta}_D)$ depends on the underlying normal distribution for the aggregated variants and may be disrupted by low-frequency variants, therefore, we restrict minor allele frequency (MAF) to at least 0.05. For details of deriving the expectations and variances under single-locus model and multiple-loci model please refer to the following two sections. The single-locus model is exact but is limited to independent variants, while the multiple-loci model is asymptotic but takes into account the global LD between m variants. Therefore, the multiple-loci model is preferred in real data analysis and has been adopted to perform subsequent relatedness inferences. Given the context of Equations 3 and 4, a negative $\widehat{\theta}_D$ may result from sampling variance but could also indicate diversity among the samples.

Variance of deepKin under the single-locus model

We derived the variance of deepKin under the context of binomial distribution. If we consider two diploid individuals, each with two alleles (A and a) at one locus. The allele frequencies are p and q for allele A and a, respectively. For a pair of relatives, the probabilities of two alleles conditioning on the probability of relatedness θ is:

Allelic pair for relatives	Probability
A/A	$\theta p + (1-\theta)p^2$
A/a	$(1-\theta)pq$
a/A	$(1-\theta)pq$
a/a	$\theta q + (1-\theta)q^2$

The above table can be applied to their respect second allele pair. The probabilities of sixteen genotype combinations between two individuals are filled in this 4×4 table, which is symmetric.

		First allelic pair			
	**Probability**	A/A	A/a	a/A	a/a
Second allelic pair	A/A	$[\theta p+(1-\theta)p^2]^2$	$(1-\theta)pq$ $[\theta p+(1-\theta)p^2]$	$(1-\theta)pq$ $[\theta p+(1-\theta)p^2]$	$[\theta p+(1-\theta)p^2]$ $[\theta q+(1-\theta)q^2]$
	A/a		$(1-\theta)^2p^2q^2$	$(1-\theta)^2p^2q^2$	$(1-\theta)pq$ $[\theta q+(1-\theta)q^2]$
	a/A			$(1-\theta)^2p^2q^2$	$(1-\theta)pq$ $[\theta q+(1-\theta)q^2]$
	a/a	Symmetric			$[\theta q+(1-\theta)q^2]^2$

Ignoring the order of two individuals, we can recalculate the frequencies by summing up the corresponding probabilities in each cell. As individual genotypes are standardized by $\widehat{x} = \frac{x-2\widehat{p}}{\sqrt{2\widehat{pq}}}$, where x is coded as the number of minor alleles. deepKin score between individual i and individual j is estimated by $1-\frac{1}{2}\left(\frac{x_i-2\widehat{p}}{\sqrt{2\widehat{pq}}}-\frac{x_j-2\widehat{p}}{\sqrt{2\widehat{pq}}}\right)^2$. $\widehat{p}$ and $\widehat{q}$ are the estimated allele frequencies of A and a. The rearranged probabilities and relatedness scores are:

Genotype pair	Genotype code	Probability (f_i)	Relatedness (X_i)
$\{AA,AA\}$	$\{2,2\}$	$[\theta p+(1-\theta)p^2]^2$	1
$\{Aa,Aa\}$	$\{1,1\}$	$4(1-\theta)^2p^2q^2+2\theta pq$	1
$\{aa,aa\}$	$\{0,0\}$	$[\theta q+(1-\theta)q^2]^2$	1
$\{AA,Aa\}$	$\{2,1\}$	$4\theta(1-\theta)p^2q+4(1-\theta)^2p^3q$	$\frac{4pq-1}{4pq}$
$\{Aa,aa\}$	$\{1,0\}$	$4\theta(1-\theta)pq^2+4(1-\theta)^2pq^3$	$\frac{4pq-1}{4pq}$
$\{AA,aa\}$	$\{2,0\}$	$2(1-\theta)^2p^2q^2$	$\frac{pq-1}{pq}$

Thus, the expectation is $E(\widehat{\theta}_D) = \sum X_i f_i = \theta$ and the variance is $var(\widehat{\theta}_D) = \sum X_i^2 f_i - \sum X_i f_i = \frac{1}{2}(1-\theta)^2 + \frac{1-\theta}{4pq}$. This sampling variance is nearly exact but hardly takes LD into account, therefore, we move to the multiple-loci model.

Variance of deepKin under the multiple-loci model

The expectation and variance for deepKin ($\widehat{\theta}_D$) are,

$$E(\widehat{\theta}_D) = 1-\frac{1}{2}\frac{1}{m}E\left[\sum_l^m(\tilde{x}_{il}-\tilde{x}_{jl})^2\right] = 1-\frac{1}{2}\left[E(\tilde{x}_{il}^2)+E\left(\tilde{x}_{jl}^2\right)-2E(\tilde{x}_{il}\tilde{x}_{jl})\right] = \theta$$

$$var(\widehat{\theta}_D) = \frac{1}{4m^2}var\left[\sum_l^m(\tilde{x}_{il}-\tilde{x}_{jl})^2\right] = \frac{1}{4m^2}\left\{E\left[\left(\sum_l^m(\tilde{x}_{il}-\tilde{x}_{jl})^2\right)^2\right]-E^2\left[\sum_l^m(\tilde{x}_{il}-\tilde{x}_{jl})^2\right]\right\}$$

The first term of $var(\widehat{\theta}_D)$ can be decomposed as

$$E\left[\left(\sum_l^m(\tilde{x}_{il}-\tilde{x}_{jl})^2\right)^2\right] = E\left[\left(\sum_l^m\tilde{x}_{il}^2+\sum_l^m\tilde{x}_{jl}^2-2\sum_l^m\tilde{x}_{il}\tilde{x}_{jl}\right)^2\right]$$

$$= E\left[\left(\sum_l^m\tilde{x}_{il}^2\right)^2+\left(\sum_l^m\tilde{x}_{jl}^2\right)^2+4\left(\sum_l^m\tilde{x}_{il}\tilde{x}_{jl}\right)^2+2\sum_l^m\tilde{x}_{il}^2\sum_l^m\tilde{x}_{jl}^2-4\sum_l^m\tilde{x}_{il}^2\sum_l^m\tilde{x}_{il}\tilde{x}_{jl}-4\sum_l^m\tilde{x}_{jl}^2\sum_l^m\tilde{x}_{il}\tilde{x}_{jl}\right]$$

Each term has its expectation calculated as

Term	Expectation
$E[(\sum_l^m \tilde{x}_{il}^2)^2]$ or $E[(\sum_l^m \tilde{x}_{jl}^2)^2]$	$m^2+2m+2\sum_l^m \sum_{l'\neq l}^m \rho_{ll'}^2$
$E[(\sum_l^m \tilde{x}_{il}\tilde{x}_{jl})^2]$	$m+m\theta^2+m^2\theta^2+(1+\theta^2)\sum_l^m \sum_{l'\neq l}^m \rho_{ll'}^2$
$E(\sum_l^m \tilde{x}_{il}^2 \sum_l^m \tilde{x}_{jl}^2)$	$2m\theta^2+m^2+2\theta^2\sum_l^m \sum_{l'\neq l}^m \rho_{ll'}^2$
$E(\sum_l^m \tilde{x}_{il}^2 \sum_l^m \tilde{x}_{il}\tilde{x}_{jl})$ or $E(\sum_l^m \tilde{x}_{jl}^2 \sum_l^m \tilde{x}_{il}\tilde{x}_{jl})$	$2m\theta+m^2\theta+2\theta\sum_l^m \sum_{l'\neq l}^m \rho_{ll'}^2$

The second term of $var(\widehat{\theta}_D)$ is $E^2[\sum_l^m (\tilde{x}_{il}-\tilde{x}_{jl})^2] = 4m^2(1-\theta)^2$.
Therefore,

$$var(\widehat{\theta}_D) = \frac{1}{4m^2}\left[2m^2+4m+4\sum_l^m\sum_{l'\neq l}^m \rho_{ll'}^2 + 4m + 4m\theta^2 + 4m^2\theta^2 + 4(1+\theta^2)\sum_l^m\sum_{l'\neq l}^m \rho_{ll'}^2 + 4m\theta^2 + 2m^2 \right.$$
$$\left. + 4\theta^2\sum_l^m\sum_{l'\neq l}^m \rho_{ll'}^2 - 16m\theta - 8m^2\theta - 16\theta\sum_l^m\sum_{l'\neq l}^m \rho_{ll'}^2 - 4m^2(1-\theta)^2\right] = \frac{1}{4m^2}\left[8m - 16m\theta + 8m\theta^2\right.$$
$$\left. + 8(1-\theta)^2\sum_l^m\sum_{l'\neq l}^m \rho_{ll'}^2\right] = \frac{1}{4m^2}\left[8m(1-\theta)^2 + 8(1-\theta)^2\left(\frac{m^2}{m_e}-m\right)\right] = \frac{2(1-\theta)^2}{m_e}$$

Relatedness inference: Deepest significant estimation

The deepKin's framework provides statistical inference for the estimated relatedness score. To test whether a pair of individuals is related, the null distribution of unrelated pairs is $N\left(0,\frac{2}{m_e}\right)$ given by Equation 4. Therefore, the Z score and corresponding p-value on $\widehat{\theta}_D$ are $Z = \widehat{\theta}_D \Big/ \sqrt{\frac{2}{m_e}} \sim N(0,1)$ and $p = 1-\phi(Z)$. ϕ is the standard normal cumulative distribution function. If we assume a significant level of α, the critical value (θ_D^δ) is

$$P\left(Z = \frac{\widehat{\theta}_D}{\sqrt{\frac{2}{m_e}}} > \frac{\theta_D^\delta}{\sqrt{\frac{2}{m_e}}}\right) = \alpha$$

$$\theta_D^\delta = z_{1-\alpha}\sqrt{2/m_e} \quad \text{(Equation 5)}$$

The critical value is considered as the deepest significant estimation and $\delta = \log_{\frac{1}{2}}(z_{1-\alpha}\sqrt{2/m_e})$ will be the deepest significant degree by log-transformation from relatedness to relationship degree.

Relatedness inference: Classification

Although the relatedness score can be continuous, we classify them into discrete classes for easier access, as often done in real data analysis. Since Equation 4 gives the sampling variance for any relatedness, we can make a concrete inference for any observed significant relatedness between t and $t+1$ degree using hypothesis testing. The null distributions of the relatedness estimations at t and $t+1$ degrees are $N\left(\theta^t, \frac{2(1-\theta^t)^2}{m_e}\right)$ and $N\left(\theta^{t+1}, \frac{2(1-\theta^{t+1})^2}{m_e}\right)$ respectively. According to definition, we have $\theta^t = \frac{1}{2^t}$ and $\theta^{t+1} = \frac{1}{2^{t+1}}$. The classification of x degree to t or $t+1$ degree is performed based on two z-test statistics. For hypothesis H_0: $\theta^x = \theta^t$, H_1: $\theta^x < \theta^t$, we can calculate its Z score and p-value of rejecting H_0 as

$$Z_t^* = \frac{\theta^x - \theta^t}{\sigma_{\theta^t}} = \frac{\left|\frac{1}{2^x}-\frac{1}{2^t}\right|}{\sqrt{\frac{2\left(1-\frac{1}{2^t}\right)^2}{m_e}}} \sim N(0,1), p_t = 1-\phi(Z_t^*)$$

For hypothesis H_0: $\theta^x = \theta^{t+1}$, H_1: $\theta^x > \theta^{t+1}$, we can calculate its Z score and p-value of rejecting H_0 as

$$Z_{t+1}^* = \frac{\theta^x - \theta^{t+1}}{\sigma_{\theta^{t+1}}} = \frac{\left|\frac{1}{2^x} - \frac{1}{2^{t+1}}\right|}{\sqrt{\frac{2\left(1 - \frac{1}{2^{t+1}}\right)^2}{m_e}}} \sim N(0,1), p_{t+1} = 1 - \phi\left(Z_{t+1}^*\right)$$

By comparing these two p-values, we are able to infer the classification for any observed θ^x. However, it is worth noting that in some cases, both null hypotheses may be rejected when both p-values are significant. Intuitively, there exists a specific point of relatedness where the two p-values are equal. At this crossover point for p-values, the relatedness value can be utilized as the boundary for direct classification. Let $Z_t^* = Z_{t+1}^*$, when $t < x < t+1$, we have

$$\frac{\frac{1}{2^t} - \frac{1}{2^x}}{\sqrt{\frac{2\left(1 - \frac{1}{2^t}\right)^2}{m_e}}} = \frac{\frac{1}{2^x} - \frac{1}{2^{t+1}}}{\sqrt{\frac{2\left(1 - \frac{1}{2^{t+1}}\right)^2}{m_e}}}$$

$$\frac{1}{2^x} = \frac{\frac{1}{2^t}\left[3 - 2\frac{1}{2^t}\right]}{4 - 3\frac{1}{2^t}} = \frac{3 \times 2^{t-1} - 1}{2^{2t+1} - 3 \times 2^{t-1}}$$

We compare the variance-based lower boundary with the traditional geometric mean-based lower boundary.

When $t = 0$, the sampling variance becomes 0, but we cannot use threshold = 1 to separate first-degree and identical pairs. Therefore, when x is between 0 and 1, we set $t = 0.1$, and the corresponding threshold becomes 0.881.

Degree	Geometric mean-based lower boundary	DeepKin variance-based lower boundary
0 (0.1)	0.707	0.881
1	0.354	0.400
2	0.177	0.192
3	0.088	0.095
4	0.044	0.047
5	0.022	0.024
…	…	…
t	$\frac{1}{2^{(2t+1)/2}}$	$\frac{3 \times 2^{t-1} - 1}{2^{2t+1} - 3 \times 2^{t-1}}$

Derived guidelines from deepKin

Two parameters are involved in the estimation of relatedness with deepKin: the sample size (n) and the number of markers (m). However, it is actually m_e rather than m that acts as an indicative parameter in relatedness inference, affecting the performance of deepKin. Making relatedness inference of $n(n-1)/2$ pairs of samples is a decision-making process. Our subsequent presentation follows the framework of power calculation, which aims to consider Type-I (α) and Type-II (β) error rates and offer preliminary yet practical guidelines that can be applied.

Guideline I: Thrifty choice for markers conditional on a target degree

Based on the expectation and variance of deepKin estimator under multiple-loci model, we can estimate the minimum m_e that is required for detecting the target degree (t) of relatedness ($\theta_D^t = \left(\frac{1}{2}\right)^t$) from unrelated pairs, which is related to Type-I and Type-II error rates (α and β),

$$m_e \geq 2\left[\frac{z_{1-\alpha} + z_{1-\beta}\left(1 - \theta_D^t\right)}{\theta_D^t}\right]^2 \quad \text{(Equation 6)}$$

Here, $z_{1-\alpha}$ and $z_{1-\beta}$ are the quantiles from the standard normal distribution. In particular, we set α under experiment-wise control after Bonferroni correction, the corresponding α is upon the total comparisons N. We briefly describe the derivation of Equation 6 based on the framework of power calculation. For hypothesis on a statistic T, which follows $T \sim N(\mu_0, \sigma_0^2)$ for H_0 and

follows $T \sim N(\mu_1, \sigma_1^2)$ for H_1. We assume that $\sigma_0^2 = f_0^2/n$ and $\sigma_1^2 = f_1^2/n$, where n is the number of sample size. For one-tailed hypothesis that $H_0 : \mu = \mu_0$, and $H_1 : \mu > \mu_0$, the minimal number of n is $\left(\frac{z_{1-\alpha}f_0 + z_{1-\beta}f_1}{\mu_1 - \mu_0}\right)^2$. Equation 6 can be derived by replacing $H_0 : \mu_0 = \theta_D^t = 0$, $H_1 : \mu_1 = \theta_D^t > 0$, $\sigma_0^2 = \frac{2}{m_e}$ and $\sigma_1^2 = \frac{2(1-\theta_D^t)^2}{m_e}$. We give a concrete numerical example for a simple illustration. Suppose that we have 500,000 individuals, which generates $N \approx 1.25 \times 10^{11}$ pairs of comparisons. We wanted to detect relatives up to the third-degree from unrelated pairs at Type I error rate of $\alpha = 0.05/N$ ($z_{1-\alpha} = 7.161$) and Type II error rate of $\beta = 0.1$ ($z_{1-\beta} = 2.326$), and the number of m_e based on Equation 6 should be no less than 8,780. Then a thrifty choice of a variant set can greatly reduce the financial cost of genotyping and computation. This gives the first guideline that selects only a subset of the genetic variants with the required m_e is sufficient for detecting certain degree of relatedness in N comparisons. Consequently, m_e/m reflects the balance between statistical power and computational cost though economical choice for markers.

Guideline II: The statistical power conditional on target degree and m_e

As often α is fixed in a study – such as after Bonferroni correction, we are able to determine how much of the power (π) could be compromised or improved for any target relatedness (θ_D^t),

$$\pi = 1 - \beta = z^{-1}\left(\frac{\sqrt{\frac{m_e}{2}}\theta_D^t - z_{1-\alpha}}{1 - \theta_D^t}\right) \quad \text{(Equation 7)}$$

For the same example above, if the target degree is 5, the power increases to 0.925; and if the target degree is 6, the power is rather reduced to 0.002. To increase statistic power, an applicable way is to increase the effective number of markers. When the effective number of markers increases, the power to detect the target relatedness increases (Figure S5). For more real data examples please refer to Table S4.[13,17]

On the effective number of markers (m_e)

The above analysis relies on the effective number of markers m_e, which is defined as $m_e = m^2 / \sum_{l_1,l_2}^m \rho_{l_1 l_2}^2$ so as to describe the average LD between genome-wide variants.[18–21] Intuitive m_e can be directly calculated through calculating all squared correlations between variant pairs (such as –r2 command in PLINK[14]), but the computational cost is $\mathcal{O}(nm^2)$ and it soon becomes unaffordable when n and especially, m, is large. However, estimating m_e by direct calculation can be computationally substantial, whilst m_e is needed as a hyperparameter in determining the potential of the data such as characterized by Equations 5, 6, and 7. Here, we propose a pair of estimators for m_e.

Estimator I: GRM-based estimator

We first introduce the GRM-based estimator below

$$\widehat{m}_e = \frac{1}{var(\mathbf{G}_{off})} \quad \text{(Equation 8)}$$

in which $\mathbf{G}_{off}$ refers to the off-diagonal elements of GRM, namely GRM-based estimator in the following context.[19] $\widehat{m}_e$ estimated from Equation 8 is asymptotically normal with its sampling variance of $\frac{4m_e^2}{n^2}$. The computational cost is $\mathcal{O}(n^2 m)$, which is often smaller than $\mathcal{O}(nm^2)$. A more detailed investigation on the relationship between genome-wide LD and GRM-based m_e estimator can be found in Huang et al. (2023).[15]

Estimator II: Randomization-based estimator

The second estimator is based on a randomization-based algorithm, which reduces the computational cost of estimating m_e from $\mathcal{O}(n^2 m)$ to $\mathcal{O}(nmB)$. Here, B is the number of iterations and is practically sufficient if $B > 100$. The randomization-based method uses B random vectors $\mathbf{z}_b$ ($b = 1,2,\ldots,B$), whose elements are randomly sampled from the standard normal distribution. By calculating a statistic $L_B = \frac{1}{Bm^2}\sum_{b=1}^{B}\mathbf{z}_b^T\tilde{\mathbf{X}}\tilde{\mathbf{X}}^T\tilde{\mathbf{X}}\tilde{\mathbf{X}}^T\mathbf{z}_b$, where $\tilde{\mathbf{X}}$ is the standardized genotype matrix, the empirical randomization-based estimator is then $\widehat{m}_e = \frac{n(n+1)}{L_B - n}$. Its asymptotic sampling variance is $var(\widehat{m}_e) \approx \frac{\widehat{m}_e^4}{n^4}\sigma_{L_B}^2$, in which $\sigma_{L_B}^2$ can be estimated from the B rounds of iteration for the estimation of L_B. The randomized estimation is inspired by randomized estimation of heritability,[22] but the computational technique is formally known as Girard-Hutchison estimation.[23,24]

In the simulations below, m_e was estimated using GRM-based estimator, while in the 3K Oxford subset and 430K White British subset below, m_e were estimated using randomization-based estimator. More details about m_e please refer to Methods S1. The validity of the randomization-based estimator was confirmed using 3K Oxford subset (Figure S1A). Even though GRM will be calculated during deepKin estimation and consequently m_e could be estimated, the randomization-based estimator still has its advantages of a faster approximation thus a faster evaluation of the guidelines, which promises a faster decision on SNP set selection.

Simulation

Variance validation

We first validated the single-locus model (Equation 3). $n = 2{,}000$ pairs of individuals with relatedness score θ were simulated, and $\theta = 0.5, 0.25, 0.125$, and 0, respectively. 5,000 equal-frequency markers were simulated, and the frequency were of 0.01, 0.05, 0.10, 0.15, 0.20, 0.25, 0.30, and 0.35, respectively. Both expected and observed variances were evaluated among 2,000 pairs of individuals. Standard deviation of each variance estimation was evaluated through ten repeats.

To validate Equation 4 based on multiple-loci model, we simulated the same $n = 2{,}000$ pairs of individuals using 5,000 markers but whose MAFs were sampled from a uniform distribution, which were $U(0.01,0.5)$, $U(0.05,0.5)$, $U(0.10,0.5)$, $U(0.15,0.5)$, $U(0.20,0.5)$, $U(0.25,0.5)$, $U(0.30,0.5)$, and $U(0.35,0.5)$, respectively. We used D' (Lewontin's measure) to represent the LD, which was sampled from a uniform distribution $U(0.1, 0.2)$ and $U(0.5, 0.8)$ to represent low and high LD conditions. m_e that were employed to calculate the asymptotic variance was estimated from GRM-based estimator. Ten repeats were used to evaluate the standard deviation of each variance estimation.

Simulation for a pair of biallelic loci in LD

D is the commonly used gametic disequilibrium parameter, and D has its upper bound and lower bound upon to the allele frequencies. However, it would be easier to simulate a pair of loci given D' (Lewontin's measure), the relative gametic disequilibrium as defined by Lewontin, the value of which is between -1 and 1. Let the allele frequencies be p_A and q_A for allele A (coded 1) and a (coded 0) of the first locus, p_B and q_B for allele B (coded 1) and b (coded 0) of the second locus. The frequencies of the four haplotypes of these two loci are p_{AB} (haplotype AB), p_{Ab} (haplotype Ab), p_{aB} (haplotype aB), and p_{ab} (haplotype ab). D' can be specified for a pair of loci in question,

$$D' = \begin{cases} \dfrac{D}{\min\left(p_A q_B, q_A p_B\right)}, D > 0 \\ \dfrac{D}{\min\left(p_A p_B, q_A q_B\right)}, D < 0 \end{cases}$$

in which $D = p_{AB} - p_A p_B$. In simulation, under random mating, the conditional probability of generating the second locus could be expressed as,

$$P(L_B = 1|L_A = 1) = \frac{P(L_B = 1, L_A = 1)}{p_A} = \frac{p_A p_B + D}{p_A}$$

$$P(L_B = 0|L_A = 1) = \frac{P(L_B = 0, L_A = 1)}{p_A} = \frac{p_A q_B - D}{p_A}$$

$$P(L_B = 1|L_A = 0) = \frac{P(L_B = 1, L_A = 0)}{q_A} = \frac{q_A p_B - D}{q_A}$$

$$P(L_B = 0|L_A = 0) = \frac{P(L_B = 0, L_A = 0)}{q_A} = \frac{q_A q_B + D}{q_A}$$

Data-based threshold determination

We showed the difference in the inference frameworks between KING and deepKin in simulation. Only unrelated individuals ($n = 2{,}000$) were simulated based on different number of markers ($m = 1{,}000$, 5,000, 10,000, and 50,000). The genotype simulation followed the LD scenario as described above. MAF was sampled from a uniform distribution $U(0.05, 0.5)$ and D' was sampled from a uniform distribution $U(0.1,0.2)$, and correspondingly $m_e = 978$, 4,896, 9,782, and 48,967 as estimated by the GRM-based estimator. We applied the KING's inference criteria of 0.707 (the geometric mean of 1 and 0.5) for monozygotic twins and duplicate samples, 0.354, 0.177, 0.088, and 0.044 for first-, second-, third-, and fourth-degree of relatedness, respectively. We calculated the deepest significant relatedness estimation (θ^{δ}) from Equation 5 based on m_e at the significant level of $\alpha = 0.05/1{,}999{,}000$. This enables data-specific thresholds for relatedness inference.

Relatedness inference based on p-values

To validate the role of p-values in inferring relatedness, we simulated various related pairs in two cohorts. We simulated 200 individuals each for cohort 1 and cohort 2 ($n_1 = n_2 = 200$). Between cohort 1 and cohort 2, we generated 10 pairs of related samples up to the fourth-degree. MAF was sampled from a uniform distribution $U(0.05, 0.5)$ and D' was sampled from a uniform distribution $U(0.1, 0.2)$. The numbers of markers were $m = 133$, 687, 3,086, 13,050, and 53,643, corresponding to identical, first-, second-, third-, and fourth-degree relatives, respectively. Taking fourth-degree as an example, the number of markers was determined by the following procedures. the minimum number of m_e based on Equation 6 for fourth-degree was 17,881 at Type I error rate of $\alpha = 0.05/40{,}000$ and Type II error rate of $\beta = 0.1$. To make sure that the actual m_e of simulated markers meets the requirement, we simulated m markers that are three times of the minimum m_e, $m = 3m_e = 53{,}643$. The actual m_e of 53,643 markers were 18,612. All sets of selected markers

met the requirement of the target minimum number of m_e. m_e was estimated from GRM-based estimator. The deepest significant relatedness estimations were calculated according to m_e based on Equation 5 at a significant level of 0.05/40,000.

UK Biobank application

Oxford demo for the proof of principle

We drew 3,000 UK Biobank (UKB) participants from Oxford and analyzed four sets of SNPs, each representing a different effective number of markers. The first set had 693,666 imputation loci after QC, and the distribution of MAF was as shown in Figure S6A. The inclusion criteria for autosome variants were: i) MAF >0.05; ii) Hardy-Weinberg equilibrium (HWE) test p-value > 1e-7; iii) no locus missingness. The second SNP set had the same inclusion criteria as SNP set 1, but the MAF threshold was increased from 0.05 to 0.2 and resulted in 298,221 SNPs. The third SNP set excluded variants that had high population differentiation in SNP Set 2, which remained 237,642 variants and were often called as non-ancestry informative markers (non-AIMs).[25] The fourth SNP set was performed LD pruning on SNP set 2 (r^2 <0.1 in a 50-variant window and a 5-variant count to shift the window), which reduced the number of variants to 36,425.

We estimated m_e for each of the four SNP sets using the randomization-based method. To further describe how the choice of the SNP sets could result in the fluctuation of m_e, we examined a total of 13 conditions, 6 of which were randomly selected markers with different sizes (m = 5,000, 10,000, 50,000, 100,000, 150,000, and 200,000) and 7 of which had different pruning thresholds ($r^2 < 0.05$, 0.1, 0.2, 0.3, 0.4, 0.5, and 0.8 in a 50-variant window and a 5-variant count to shift the window). All conditions were performed on SNP set 2.

The value of m_e were further tested through relatedness estimation based on two genome regions with extreme LD conditions. We have selected two genome regions, Region I (specifically from the centromere region of chromosome 11, 46.06–59.41 Mb) and Region II (chromosome 1, 0.75–33.45 Mb). Both two regions included 7,974 QCed SNPs (MAF>0.05). We used X-LD to calculate LD in blocks with 200 SNPs per block[15] and exhibited in heatmaps. Relatedness estimation were performed by deepKin, where m_e, expected and observed variances were calculated.

Relatedness in UKB White British ancestry subset

We considered a subset of 427,287 participants with self-reported White British ancestry from 19 assessment centers with a sample size greater than 5,000. Leeds had the largest sample size of 39,707, while Bury had the smallest sample size of 7,701. 72,016 imputation SNPs remained after QC (Figure S6B). The inclusion criteria for autosome variants were: i) MAF >0.05; ii) HWE test p-value > 1e-7; iii) no locus missingness; iv) r^2 <0.1 in a 50-variant window and a 5-variant count to shift the window. To explore the geographical connection between relatives, we included the grid co-ordinates for 19 assessment centers, which were downloaded from the UKB website (https://biobank.ndph.ox.ac.uk/ukb/refer.cgi?id=11002).

QUANTIFICATION AND STATISTICAL ANALYSIS

This study presents method for relatedness inference based on statistical procedures and tests which are discussed in detail in the sections above. Further statistical tests and measures are reported in the figure legends.

Cell Reports Methods, Volume *5*

Supplemental information

Precise estimation of in-depth relatedness in biobank-scale datasets using deepKin

Qi-Xin Zhang (张齐心), Dovini Jayasinghe, Zhe Zhang (张哲), Sang Hong Lee, Hai-Ming Xu (徐海明), and Guo-Bo Chen (陈国波)

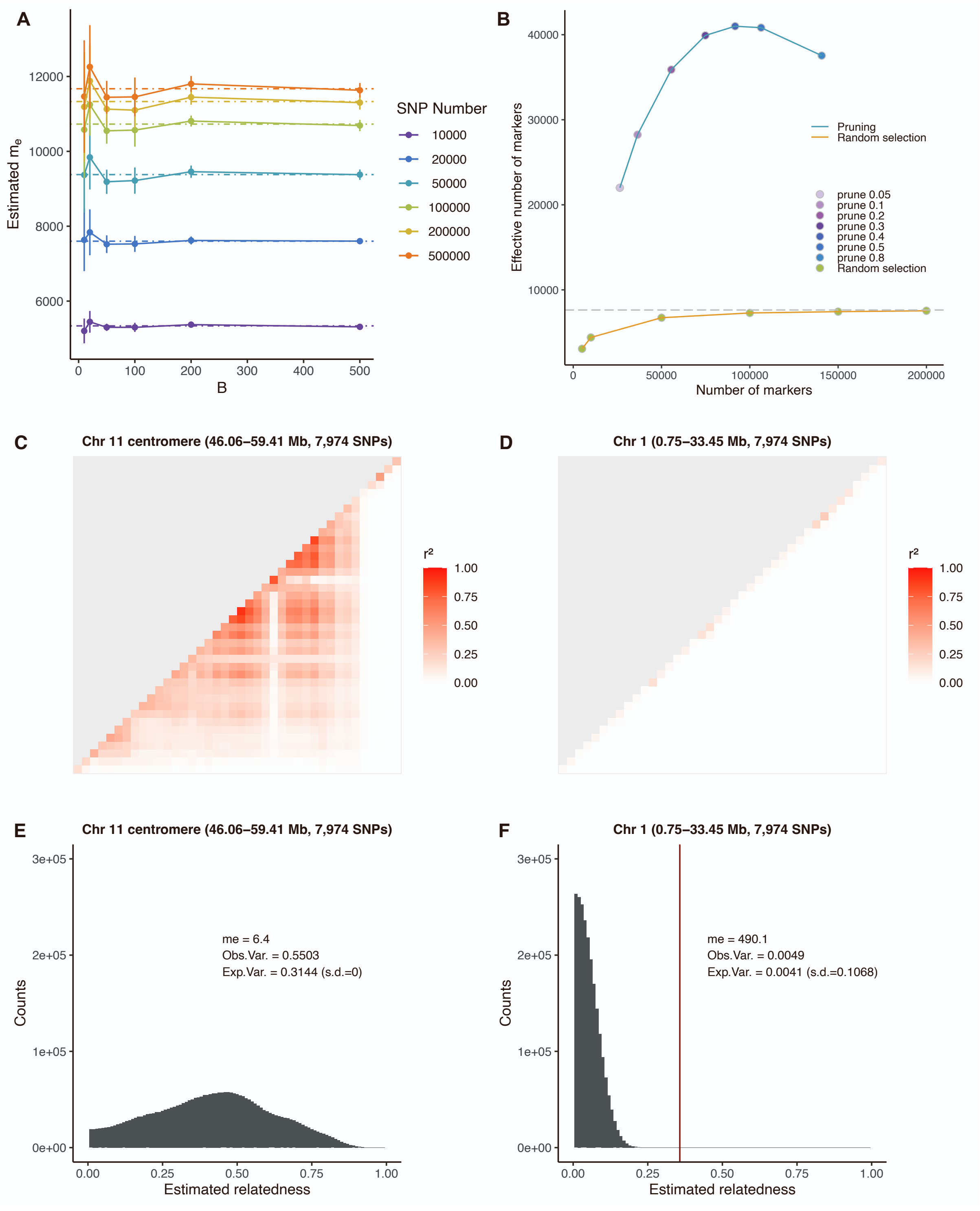

Figure S1 Estimation and evaluation on the effective number of markers (m_e) in 3K Oxford subset, related to STAR methods.

(**A**) The estimated m_e using randomization-based estimator. We have tested on 10K, 20K, 50K, 100K, 200K, and 500K SNPs and the number of iterations from 10 to 500. The dashed lines were the estimated m_e using GRM-based estimator, while each point represented the estimated m_e using randomization-based estimator. As the number of iterations increases, the randomization estimation of m_e approaches the true value.

(**B**) The distribution of the effective number of markers under different SNP sets. A total of 13 SNP sets with different QC criteria were considered, 6 of which were random selected but of different numbers (m =

5,000, 10,000, 50,000, 100,000, 150,000, and 200,000) and 7 were under different pruning thresholds ($r^2<$ 0.05, 0.1, 0.2, 0.3, 0.4, 0.5, and 0.8). The grey dashed line indicated the m_e of all m = 298,211 variants.

(**C** and **D**) LD heatmap based on two selected genome regions. LD blocks are plotted based on 200 SNP per block.

(**E** and **F**) Histogram plots on estimated relatedness by deepKin based on SNPs from two selected genome regions. Solid line indicates the threshold of significant relatedness given by deepKin.

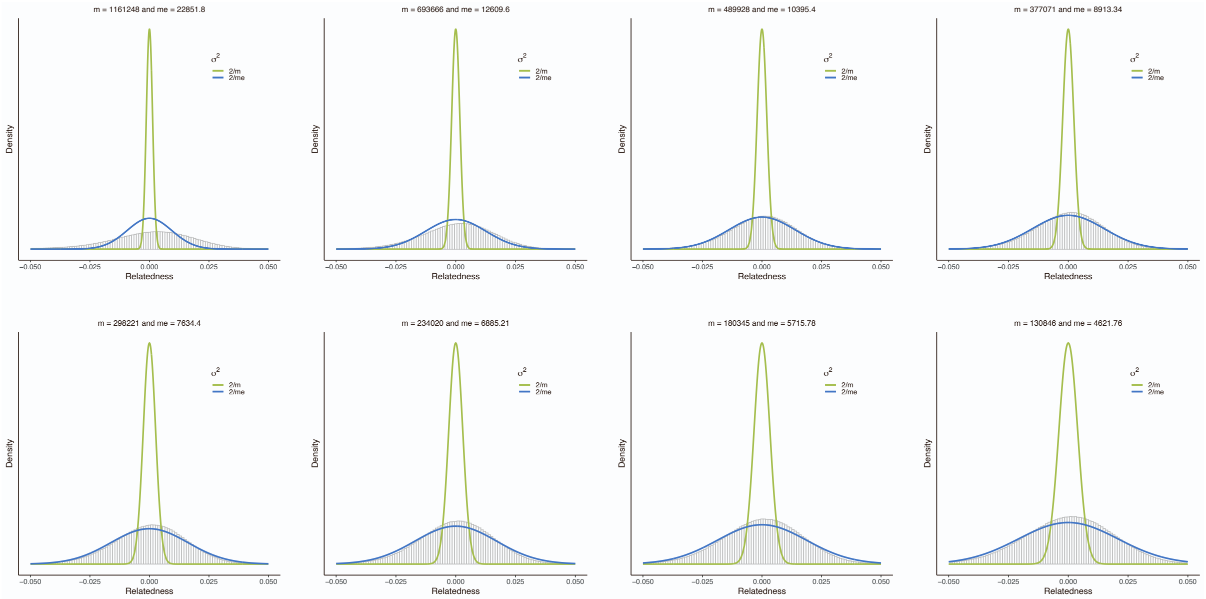

Figure S2 The histogram of estimated relatedness scores based on different MAF thresholds, related to Table 1.

MAF thresholds from left to right, from up to bottom are 0.01, 0.05, 0.10, 0.15, 0.20, 0.25, 0.30, and 0.35. The green and blue curves indicate the normal distribution of $N(0,2/m)$ and $N(0,2/m_e)$, respectively.

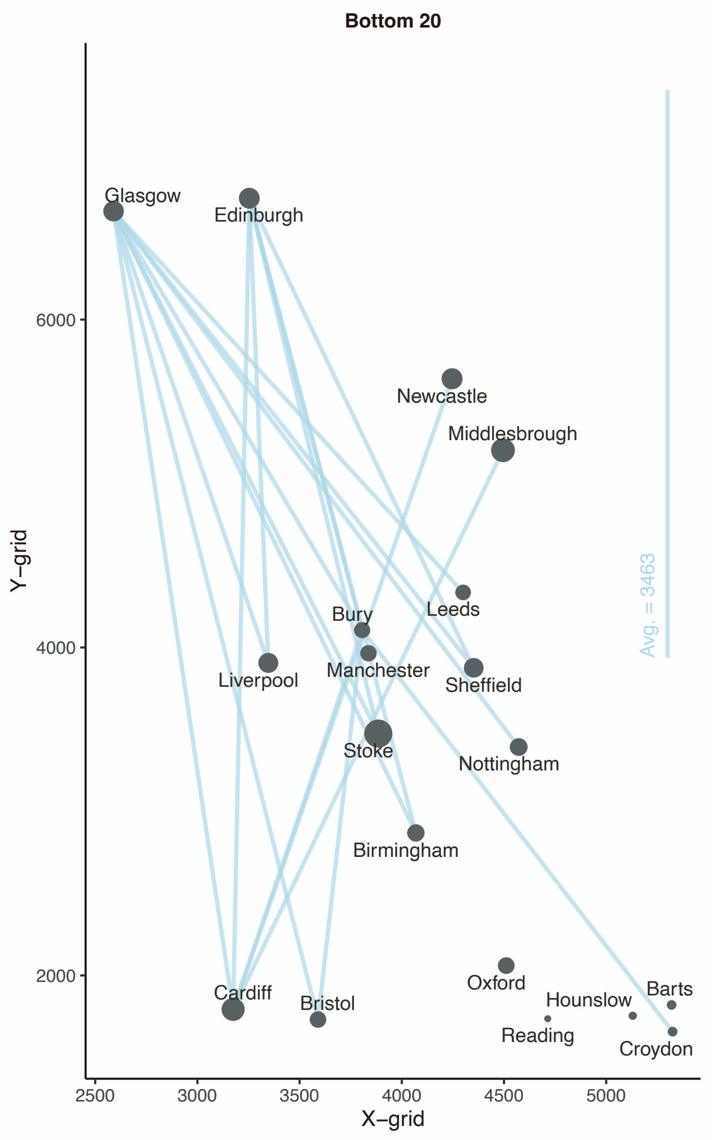

Figure S3 Grid coordinates of 19 assessment centers in UKB and the bottom 20 pair-wise proportion of cross-cohort significant relatives, related to Figure 5.

The averaging distance is calculated from the average straight-line distance of 20 pairs of cohorts in the plot. The size of the dot indicates the size of the proportion of within-cohort significant relatives.

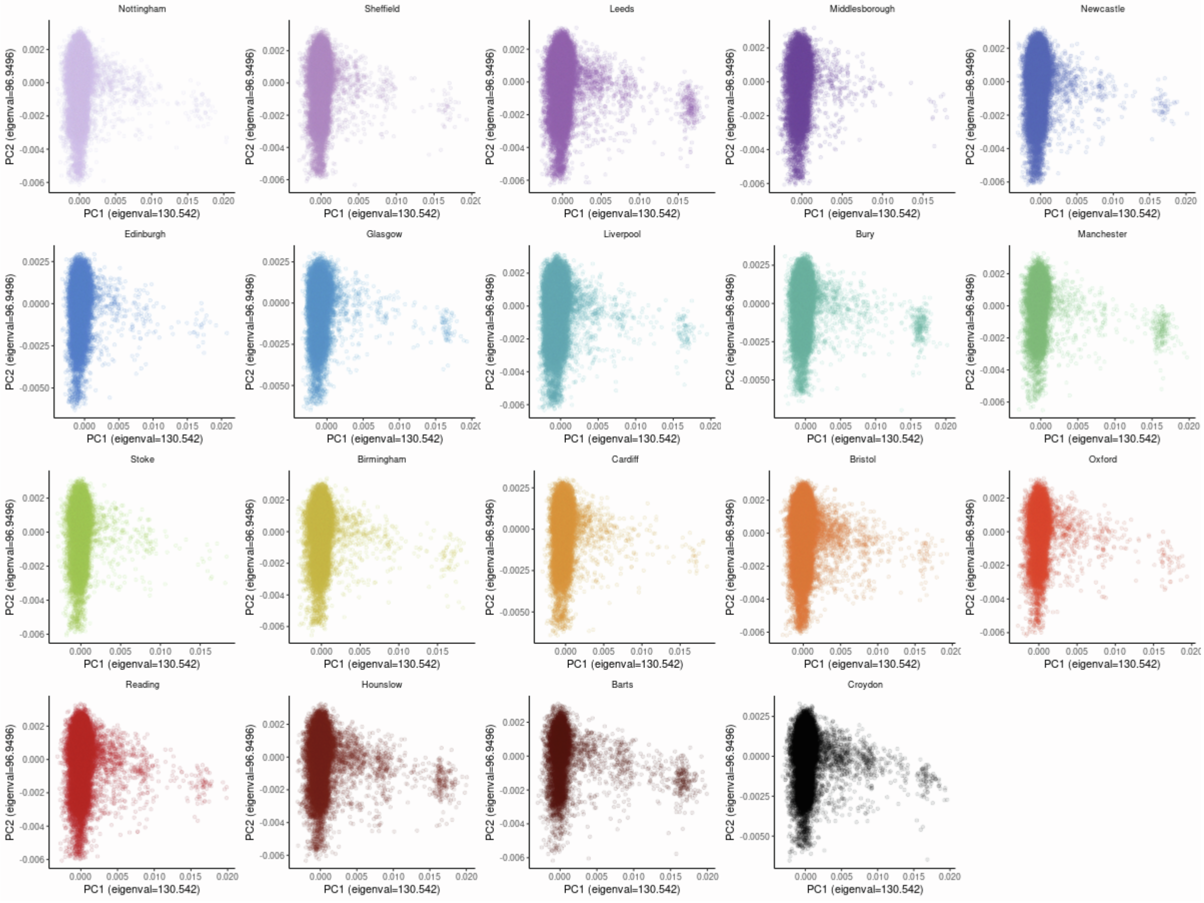

Figure S4 The distribution of the first two PCs for individuals in the 19 UKB cohorts, related to Figure 5.

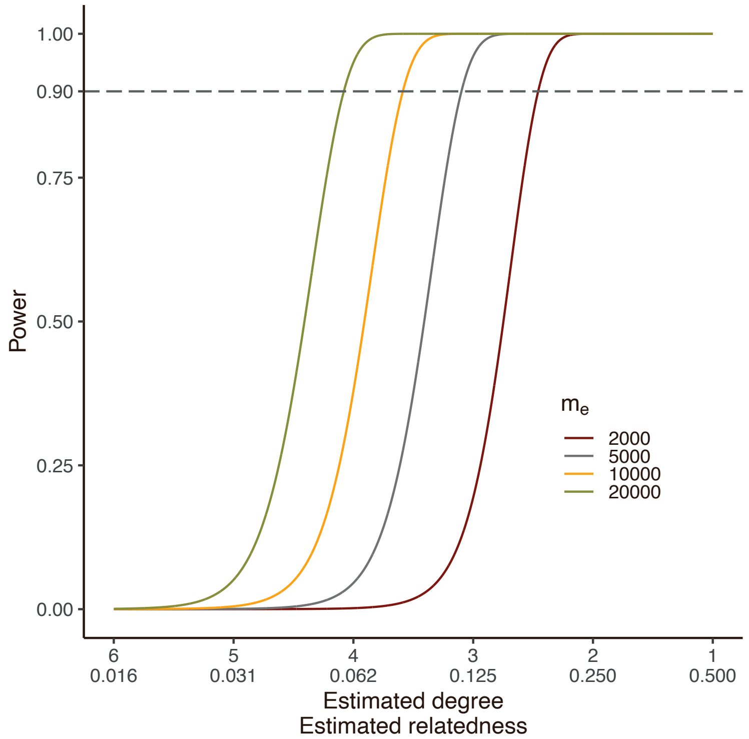

Figure S5 The power of detecting relatedness under different effective number of markers, related to STAR methods. The power is calculated based on Eq 7 under four different effective number of markers (m_e = 2,000, 5,000, 10,000, and 20,000). The dashed grey line indicates 90% power. A range of degrees from zero to six are considered. Suppose Type I error rate of $\alpha = 0.05/N$ and Type II error rate of β=0.1, where $N = 40{,}000$.

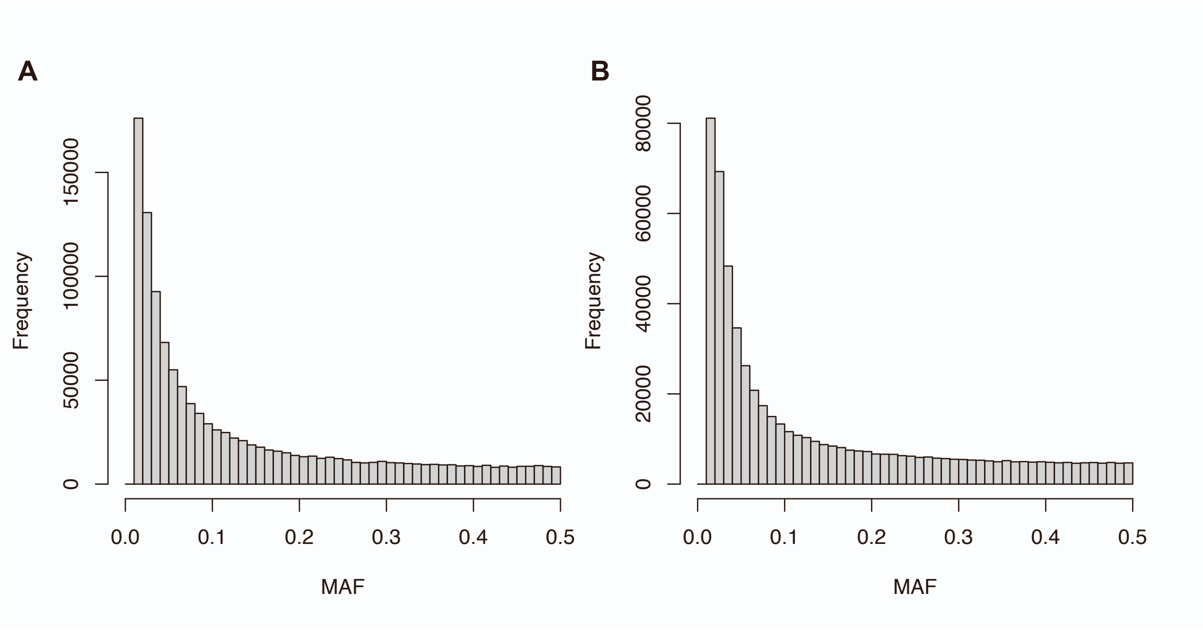

Figure S6 The histograms of MAF distribution in two UKB subsets after QC, related to STAR methods. (**A**) In 3K Oxford subset and (**B**) in 430K white British subset. QC criteria: MAF > 0.01; HWE test *p*-value > 1e-7; no locus missingness.

Table S2 QC details for four SNP sets and the numbers of pairs exceed relatedness cut-offs based on two normal GRM cutoffs in the Oxford demo, related to Table 1.

SNP set	QC details	Number of SNPs	m_e	δ	GRM Cut-off	Number of pairs
Set 1	MAF>0.05	693,666	12,610	3.827	θ>0.05	2,381
					θ>0.025	47,268
Set 2	MAF>0.2	298,221	7,634	3.465	θ>0.05	539
					θ>0.025	72,032
Set 3	MAF>0.2;	237,642	10,424	3.690	θ>0.05	108
	eigenGWAS *p*-value>0.05				θ>0.025	22,564
Set 4	MAF>0.2;	36,425	28,244	4.409	θ>0.05	60
	LD pruning (50 5 0.1)				θ>0.025	655

Table notes: 3,000 individuals and a total number of ~4.5 million comparisons are considered.

*δ is the deepest significant degree that the data would support to detect from unrelated individuals.

*m_e is estimated by randomized estimation.

Table S3 Summary of the significant related pairs in UKB white British dataset, related to Figure 5.

Degree	Identical	1st-degree	2nd-degree	3rd-degree	4th-degree	5th-degree	Total
Number of pairs	162	25,699	9,455	53,221	91,977	31,606	212,120

Table S4 Real dataset examples for applying the deepKin guidelines, related to STAR methods.

Data set	n	m_e (m)	Guideline I		Guideline II	
			θ^t	min m_e	θ^t	Power
UKB white British	427,287	56,945 (72,016)	1	101	1	1.000
			0.5	482	0.5	1.000
			0.25	2,089	0.25	1.000
			0.125	8,690	0.125	1.000
			0.0625	35,437	0.0625	1.000
			0.03125	143,116	0.03125	0.028
UKB Chinese	1,435	38,948 (238,789)	1	57	1	1.000
			0.5	285	0.5	1.000
			0.25	1,267	0.25	1.000
			0.125	5,331	0.125	1.000
			0.0625	21,855	0.0625	1.000
			0.03125	88,493	0.03125	0.158
Middle East	137	22,049 (1,321,919)	1	39	1	1.000
			0.5	203	0.5	1.000
			0.25	920	0.25	1.000
			0.125	3,905	0.125	1.000
			0.0625	16,075	0.0625	0.989
			0.03125	65,223	0.03125	0.124

Table notes: n is the number of sample size; m is the number of markers; m_e is the effective number of markers and is estimated by randomized estimation. We suppose Type I error rate of $\alpha = 0.05/N$ and Type II error rate of $\beta = 0.1$, where $N = n(n-1)/2$ The first two datasets are from UKB and the third data sets are from https://doi.org/10.1016/j.cell.2021.07.013.

Methods S1. All about m_e, related to STAR Methods

Contents

1 Introduction **2**

2 Definition of m_e **2**

3 Estimation for m_e **3**

3.1 Method I: GRM-based estimation 3

3.2 Method II: $tr(\mathbf{K}^2)$ estimation 4

4 General linkage disequilibrium and m_e **5**

1 Introduction

In both the linkage (family-based design) and the GWAS (population-based design) era, the concept of m_e is raised and developed. The concept of "independent number of segments/markers" raises in particular when genome-wide markers are involved in determining the sampling variance of population statistics, such as the sampling variance of multilocus identity by descent (IBD) score in terms of chromosomal length $m_{e.L}$. The early pioneering work relevant to $m_{e.L}$ can be found in Stam, who estimated multilocus IBD (1), and a good example of how the chromosomal length that determines the distribution of IBD for full sibs can be found in Visscher et al. (2); Hill and Weir gave a comprehensive summary of how $m_{e.L}$ determines the sampling variance for various relatives (3). If $m_{e.L}$ is related to chromosomal recombination, while in the GWAS era m_e is related to linkage disequilibrium (LD). From genomic selection (4; 5; 6), polygenic risk score (7; 8; 9), estimation of the sampling variance of SNP-heritability (h^2_{SNP}) (10; 11; 12; 13), and even in the emerging field such as differential privacy for membership inference (14), m_e is deeply involved.

2 Definition of m_e

As a continuous concept from the linkage to the GWAS era like m_e, we nevertheless only focus on its implication in GWAS here. The m_e naturally creeps in when the genetic relationship matrix (GRM) is involved. We give some typical circumstances that m_e shows in

$$\begin{cases} var(h^2_{snp}) & = & \frac{2m_e}{n^2} & (10;\ 15) \\ var(\theta) & = & \frac{1+\theta^2}{m_e} & (14) \end{cases} \tag{1}$$

$var(h^2_{SNP})$ is the sampling variance for h^2_{SNP} under REML, but it is variations of this term when the method is switched to method of moment (MoM) (11; 13). Even under its randomized application for MoM, the sampling variance the estimated h^2_{SNP} involved m_e but with further modification (16). θ is realized genetic relatedness estimated in genome-wide markers.

For a dataset of n individuals and m SNPs, GRM is defined as

$$\mathbf{K} = \frac{\mathbf{X}\mathbf{X}^T}{m} \tag{2}$$

and $\theta_{i,j}$ refers to the ith and j^{th} element in $\mathbf{K}$,

$$\theta_{i,j} = \frac{1}{m}\sum_{l}^{m} x_{i,l}x_{j,l} \tag{3}$$

A global measure of LD for m^2 pairs of markers is

$$m_e = \frac{m^2}{\sum_{l_1,l_2} r^2_{l_1 l_2}} = \frac{m^2}{m + \sum_{l_1 \neq l_2} r^2_{l_1 l_2}} \tag{4}$$

in which $r^2_{l_1 l_2}$ the squared correlation between a pair of biallelic loci l_1 and l_2 (17). Furthermore, $r^2_{l_1 l_2} =$

$\frac{D^2_{l_1l_2}}{p_{l_1}q_{l_1}p_{l_2}q_{l_2}}$, in which $D_{l_1l_2}$ is another commonly used LD metric and $q_{l_1} = 1 - p_{l_1}$ (p_{l_1} the allele frequency of the l_1^{th} locus). Obviously, when all markers are in pairwise linkage equilibrium, $m_e = m$ because of $\sum_{l_1 \neq l_2} r^2_{l_1l_2} = 0$. According to the Isserlis theorem (18), we have

$$\begin{cases} E(\theta_{ij,l_1}\theta_{ij,l_2}) & = & E(x_{il_1}x_{jl_1})E(x_{il_2}x_{jl_2}) + E(x_{il_1}x_{il_2})E(x_{jl_1}x_{jl_2}) + E(x_{il_1}x_{jl_2})E(x_{jl_1}x_{il_2}) \\ & = & \theta_{ij}r_{l_1l_1}\theta_{ij}r_{l_2l_2} + \theta_{ii}\theta_{jj}r^2_{l_1l_2} + \theta^2_{ij}r^2_{l_1l_2} = \theta^2_{ij} + (\theta_{ii}\theta_{jj} + \theta^2_{ij})r^2_{l_1l_2} \\ E(\theta_{ij,l_1}) & = & \theta_{ij}r_{l_1} \\ E(\theta_{ij,l_2}) & = & \theta_{ij}r_{l_2} \end{cases} \tag{5}$$

One kernel element is

$$\begin{cases} cov(\theta_{ij,l_1}, \theta_{ij,l_2}) & = & E(\theta_{ij,l_1}\theta_{ij,l_2}) - E(\theta_{ij,l_1})E(\theta_{ij,l_2}) \\ & = & (\theta_{ij}r_{l_1l_1}\theta_{ij}r_{l_2l_2} + \theta_{ii}\theta_{jj}r^2_{l_1l_2} + \theta^2_{ij}r^2_{l_1l_2}) - \theta_{ij}r_{l_1}\theta_{ij}r_{l_2} \\ & = & \theta_{ii}\theta_{jj}r^2_{l_1l_2} + \theta^2_{ij}r^2_{l_1l_2} \\ var(\theta_{ij,l_1}) & = & \theta_{ii}\theta_{jj}r^2_{l_1l_1} + \theta^2_{ij}r^2_{l_1l_1} \end{cases} \tag{6}$$

For unrelated individuals, $cov(\theta_{ij,l_1}, \theta_{ij,l_2}) = r^2_{l_1l_2}$ and $var(\theta_{ij}) = 1$; the correlation between θ_{ij,l_1} and θ_{ij,l_2} is of $r^2_{l_1l_2}$.

3 Estimation for m_e

From the first glance, the estimation of m_e has a computational cost of $\mathcal{O}(nm^2)$, which is infeasible if m is, unfortunately the case in GWAS, big. Almost no conventional methods tackle the estimation of m^2 pairwise LD (19; 20). So, a couple of methods have been developed to estimate m_e.

3.1 Method I: GRM-based estimation

In appendix 1 of Goddard's work (4), a method is proposed to estimate global LD for m^2 of SNPs

$$m_{e.1} = \frac{1}{var(K_o)} \tag{7}$$

in which K_o is the $\frac{n(n-1)}{2}$ off-diagonal elements in $\mathbf{K}$. Furthermore, the asymptotic distribution of $m_{e.2}$ is $N(m_e, \frac{4m_e^2}{n^2})$.

So, the mathematical expectation is (for unrelated samples $E(\theta_{ij}) = 0$)

$$\begin{aligned} var(K_o) & = E(\theta^2_{ij}) - E^2(\theta_{ij}) \\ & = \tfrac{1}{m^2}\textstyle\sum^m_{l_1,l_2}(\theta_{ii}\theta_{jj}r^2_{l_1l_2} + \theta^2_{ij}r^2_{l_1l_2}) \\ & = \tfrac{1}{m^2}\textstyle\sum^m_{l_1,l_2} r^2_{l_1l_2} \end{aligned} \tag{8}$$

, and $E(1/var(K_0)) = m_e$ is a unbiased estimate for m_e. It should be noted that the computational cost is

$O(n^2m)$, which will soon be infeasible for a large sample size.

3.2 Method II: $tr(\mathbf{K}^2)$ estimation

Under random mating, we have

$$\begin{aligned} tr(\mathbf{K}^2) &= \textstyle\sum_{i,j}^{n}\sum_{l_1,l_2}^{m}\theta_{i,j}^2 \\ &= \textstyle\sum_{i,j}^{n}\sum_{l_1,l_2}^{m} x_{i,l_1}x_{i,l_2}x_{i,l_1}x_{j,l_2} \\ &= \textstyle\sum_{i,j}^{n}\{\sum_{l}^{m} x_{i,l}^2x_{j,l}^2 + \sum_{l_1,l_2}^{m} x_{i,l_1}x_{i,l_2}x_{j,l_1}x_{j,l_2}\} \\ &= \textstyle\sum_{i,j}^{n}\{\sum_{l}^{m}[E(x_{i,l}^2)E(x_{j,l}^2) + 2E^2(x_{i,l}x_{j,l})] \\ &\quad \textstyle+\sum_{l_1\neq l_2}^{m}[E(x_{i,l_1}x_{i,l_2})E(x_{j,l_1}x_{j,l_2}) + E(x_{i,l_1}x_{j,l_1})E(x_{i,l_2}x_{j,l_2}) + E(x_{i,l_1}x_{j,l_2})E(x_{i,l_2}x_{j,l_1})]\} \\ &= \textstyle\sum_{i,j}^{n}\{\sum_{l}^{m}(\theta_{ii}\theta_{jj}r_{ll}^2 + 2\theta_{ij}^2r_{ll}^2) \\ &\quad \textstyle+\sum_{l_1\neq l_2}^{m}(\theta_{ii}\theta_{jj}r_{l_1l_2}^2 + \theta_{ij}^2r_{l_1l_1}r_{l_2l_2} + \theta_{ij}^2r_{l_1l_2}^2\} \\ &= \textstyle\sum_{i,j}^{n}\{\sum_{l}^{m}(1+2\theta_{ij}^2) + \sum_{l_1\neq l_2}^{m}(r_{l_1l_2}^2 + \theta_{ij}^2 + \theta_{ij}^2r_{l_1l_2}^2)\} \\ &= \textstyle n(n+1)\sum_{l_1,l_2}^{m} r_{l_1l_2}^2 + n \end{aligned} \tag{9}$$

For biobank-scale data, $\mathbf{K}$ is so expensive to compute, a novel estimation for m_e is obtained via a randomized algorithm as follows (21)

$$L_B = \frac{1}{Bm^2}\sum_{b=1}^{B}\mathbf{z}_b^T\mathbf{X}\mathbf{X}^T\mathbf{X}\mathbf{X}^T\mathbf{z}_b, \tag{10}$$

$\{\mathbf{z}_b : b = 1, 2, \cdots, B\}$ are vectors sampled from the standard normal distribution $N(0,1)$, and $E(L_B) = tr(\mathbf{K}^2)$. Thus, the computational time complexity is $O(nmB)$. We have a randomized estimator below

$$\begin{cases} m_{e.2} &= \frac{n(n+1)}{L_B - n} \\ \text{var}(m_{e.2}) &= \frac{2\hat{m}_e^4}{n^4}\frac{\text{tr}(\mathbf{K}^4)}{B} = \frac{\hat{m}_e^4}{n^4}\sigma_{L_B}^2 \end{cases} \tag{11}$$

Interestingly, there is a variation for Method II (supplementary page 36 in (7)). This method is a simulation algorithm that evaluates quasi-independent genomic markers in GWAS data in n individuals and m SNPs. The algorithm is described in the following.

1 Randomly assign 0 or 1 to each individual with a probability p of 0.5; it mimics a balanced case-control study.

2 Conduct a single-marker regression model for GWAS and calculate a chi-square statistic for each marker.

3 Take $CS_i = \sum_j \chi^2_{1(j)}$ for the m simulation.

Repeat steps 1-3 for P rounds and calculate the sampling variance of $V_{CS} = var(CS)$. Then, the effective number of markers is consequently defined as

$$m_{e.1} = m \times \frac{2m}{V_{CS}} \tag{12}$$

$m_{e.1}$ is interpreted as the number of independent chi-square tests, a key parameter that then simplified the simulation and analytical work for polygenic risk score (7).

4 General linkage disequilibrium and m_e

It is noticed that the global LD for all pair of markers

$$\ell_g = \frac{\sum_{l_1 l_2}^{m} r_{l_1 l_2}^2}{m^2} = \frac{1}{m_e} \tag{13}$$

Factors influencing LD can equivalently influence m_e. In Equation 6, let θ_{ij,l_1} be the relatedness score of a pair of individuals at the locus l_1^{th}. $var(\theta_{ij,l_1}) = \theta_{ii}\theta_{jj}r_{l_1 l_1}^2 + \theta_{ij}^2 r_{l_1 l_1}^2$, and $cov(\theta_{ij,l_1}, \theta_{ij,l_2}) = \theta_{ii}\theta_{jj}r_{l_1 l_2}^2 + \theta_{ij}^2 r_{l_1 l_2}^2$. The expected correlation of IBS between a pair of loci is $E(\gamma_{ij}) = \frac{(\theta_{ij}^2+1)r_{l_1 l_2}^2}{\sqrt{\theta_{ij}^2+1}\sqrt{\theta_{ij}^2+1}} = r_{l_1 l_2}^2$, which is not influenced by θ_{ij}. So, we only focus on population structure here.

The very basic equation for squared correlation r^2 can be written as (17)

$$r_{l_1 l_2}^2 = \frac{cov^2(x_{l1}, x_{l_2})}{var\,(x_{l_1})\,var(x_{l_2})} \tag{14}$$

In the absence of population structure, $r_{l_1 l_2}^2 = \frac{D_{l_1 l_2}^2}{4p_{l_1}q_{l_1}p_{l_2}q_{l_2}}$, the classic definition for LD between a pair of biallelic loci. However, in the presence of population structure, the analytical resolution for LD is unknown, but can be substantial given the subpopulations (22). Considering the l_1^{th} (l_2^{th}) locus, which is divided into $\mathcal{S}_1$ ($\mathcal{S}_2$) subpopulations, each of which is assumed to be under random mating, we could obtain the frequency distribution of the three genotypes (**Table 1**). For a pair of biallelic SNPs l_1 and l_2, the conditional probabilities of four haplotype phases ($a_{l_1}a_{l_2}$, $a_{l_1}A_{l_2}$, $A_{l_1}a_{l_2}$, and $A_{l_1}A_{l_2}$) are listed in **Table 2**, and we find the variance and covariance for the two SNPs. Ohta applied a similar treatment to the island model (23).

Genotype	$a_{l_1}a_{l_2}$	$A_{l_1}a_{l_2}$	$A_{l_1}A_{l_2}$	
Code	0	1	2	Marginal probability
Subpopulation 1	$q^2_{l_1(1)}$	$2p_{l_1(1)}q_{l_1(1)}$	$p^2_{l_1(1)}$	$w_1 = \frac{n_1}{n}$
Subpopulation 2	$q^2_{l_1(2)}$	$2p_{l_1(2)}q_{l_1(2)}$	$p^2_{l_1(2)}$	$w_2 = \frac{n_2}{n}$
$\vdots$	$\vdots$	$\vdots$	$\vdots$	$\vdots$
Subpopulation $\mathcal{S}_1$	$q^2_{l_1(\mathcal{S}_1)}$	$2p_{l_1(\mathcal{S}_1)}q_{l_1(\mathcal{S}_1)}$	$p^2_{l_1(\mathcal{S}_1)}$	$w_{\mathcal{S}_1} = \frac{n_{\mathcal{S}_1}}{n}$

Table 1: Frequency distribution of three possible genotypes at the l_1^{th} SNP in subpopulations 1, 2, till $\mathcal{S}_1$

		The l_2^{th} locus		
		a_{l_2}	A_{l_2}	Marginal probability
The l_1^{th} locus	a_{l_1}	$r_{l_1l_2} = q_{l_2} + \frac{D_{l_1l_2}}{q_{l_1}}$	$\bar{r}_{l_1l_2} = p_{l_2} - \frac{D_{l_1l_2}}{q_{l_1}}$	q_{l_1}
	A_{l_1}	$\bar{R}_{l_1l_2} = q_{l_2} - \frac{D_{l_1l_2}}{p_{l_1}}$	$R_{l_1l_2} = p_{l_2} + \frac{D_{l_1l_2}}{p_{l_1}}$	p_{l_1}
Marginal probability		q_{l_2}	p_{l_2}	

Table 2: Conditional probabilities of four haplotypes for SNPs l_1 and l_2. $r_{l_1l_2} = p(a_{l_1}|a_{l_2})$ and $R_{l_1l_2} = p(A_{l_1}|A_{l_2})$ are the conditional probabilities of the two coupling haplotypes. For the sake of convenience, we are going to use $\bar{r}_{l_1l_2} = 1 - r_{l_1l_2}$ and $\bar{R}_{l_1l_2} = 1 - R_{l_1l_2}$.

Haplotype	$a_{l_1}a_{a_2}$	$a_{l_1}A_{a_2}$	$A_{l_1}a_{a_2}$	$A_{l_1}A_{a_2}$
$a_{l_1}a_{l_2}$	$r^2_{l_1l_2}q^2_{l_1}$	$r_{l_1l_2}\bar{r}_{l_1l_2}q^2_{l_1}$	$r_{l_1l_2}\bar{r}_{l_1l_2}\ p_{l_1}q_{l_1}$	$r_{l_1l_2}R_{l_1l_2}p_{l_1}q_{l_1}$
$a_{l_1}A_{l_2}$	$r_{l_1l_2}\bar{r}_{l_1l_2}q^2_{l_1}$	$\bar{r}^2_{l_1l_2}q^2_{l_1}$	$\bar{r}_{l_1l_2}\bar{R}_{l_1l_2}p_{l_1}q_{l_1}$	$\bar{r}_{l_1l_2}R_{l_1l_2}p_{l_1}q_{l_1}$
$A_{l_1}a_{l_2}$	$\bar{R}_{l_1l_2}r_{l_1l_2}p_{l_1}q_{l_1}$	$\bar{r}_{l_1l_2}\bar{R}_{l_1l_2}\ p_{l_1}q_{l_1}$	$\bar{R}^2_{l_1l_2}p^2_{l_1}$	$\bar{R}_{l_1l_2}R_{l_1l_2}p^2_{l_1}$
$A_{l_1}A_{l_2}$	$R_{l_1l_2}r_{l_1l_2}p_{l_1}q_{l_1}$	$R_{l_1l_2}\bar{r}_{l_1l_2}p_{l_1}q_{l_1}$	$\bar{R}_{l_1l_2}R_{l_1l_2}p^2_{l_1}$	$R^2_{l_1l_2}p^2_{l_1}$

Table 3: Rearranged frequency distribution of the haplotype for a pair of biallelic SNPs l_1 and l_2

$$
\begin{cases}
\begin{cases}
E(x_{l_1}) &= \sum_{k=1}^{\mathcal{S}_1} w_k(2p^2_{l_{1(k)}} + 2p_{l_{1(k)}}q_{l_{1(k)}}) = 2\bar{p}_{l_1} \\
E(x^2_{l_1}) &= \sum_{k=1}^{\mathcal{S}_1} 2w_k p_{l_{1(k)}}q_{l_{1(k)}} + \sum_{k=1}^{\mathcal{S}_1} 4w_k p^2_{l_{1(k)}} \\
var(x_{l_1}) &= E(x^2_{l_1}) - E^2(x_{l_1}) = 2\sum_{i}^{\mathcal{S}_1} w_k[p_{l_{1(k)}}q_{l_{1(k)}} + 2(p^2_{l_{1(k)}} - \bar{p}^2_{l_1})]
\end{cases} \\
\begin{cases}
E(x_{l_2}) &= \sum_{k=1}^{\mathcal{S}_2} w_k(2p^2_{l_{2(k)}} + 2p_{l_{2(k)}}q_{l_{2(k)}}) = 2\bar{p}_{l_2} \\
E(x^2_{l_2}) &= \sum_{k=1}^{\mathcal{S}_2} 2w_k p_{l_{2(k)}}q_{l_{2(k)}} + \sum_{k=1}^{\mathcal{S}_2} 4w_k p^2_{l_{2(k)}} \\
var(x_{l_2}) &= E(x^2_{l_2}) - E^2(x_{l_2}) = 2\sum_{k}^{\mathcal{S}_2} w_k[p_{l_{2(k)}}q_{l_{2(k)}} + 2(p^2_{l_{2(k)}} - \bar{p}^2_{l_2})]
\end{cases} \\
\begin{cases}
E(x_{l_1}x_{l_2}) &= 2\sum_{k=1}^{\mathcal{S}_0} w_k D_{l_1 l_{2(k)}} + 4\sum_{k=1}^{\mathcal{S}_0} w_k p_{l_{1(k)}}q_{l_{2(k)}} \\
cov(x_{l_1}, x_{l_2}) &= E(x_{l_1}x_{l_2}) - E(x_{l_1})E(x_{l_2}) = 2\sum_{k=1}^{\mathcal{S}_0} w_k[D_{l_1 l_{2(k)}} + 2(p_{l_{1(k)}}q_{l_{2(k)}} - \bar{p}_{l_1}\bar{p}_{l_2})]
\end{cases}
\end{cases}
\tag{15}
$$

These terms are sufficient to derive the general expression for LD. We plug the variance and covariance (**Equation 15**) into **Equation 14** and can further deduce a general expression for $\rho^2_{l_1 l_2}$. For simplicity, let $\mathcal{S}_0 = \mathcal{S}_1 = \mathcal{S}_2$, and we have

$$
\tilde{r}^2_{l_1 l_2} = \frac{\{\sum_{k=1}^{\mathcal{S}_0} w_k[D_{l_1 l_{2(k)}} + 2(p_{l_{1(k)}}p_{l_{2(k)}} - \bar{p}_{l_1}\bar{p}_{l_2})]\}^2}{\{\sum_{k=1}^{\mathcal{S}_0} w_k[p_{l_{1(k)}}q_{l_{1(k)}} + 2(p^2_{l_{1(k)}} - \bar{p}^2_{l_1})]\}\{\sum_{k=1}^{\mathcal{S}_0} w_k[p_{l_{2(k)}}q_{l_{2(k)}} + 2(p^2_{l_{2(k)}} - \bar{p}^2_{l_2})]\}}
\tag{16}
$$

When $\mathcal{S}_0 = 1$, $r^2_{l_1 l_2} = \frac{D^2_{l_1 l_2}}{p_{l_1}q_{l_1}p_{l_2}q_{l_2}}$, which is reduced to the conventional expression for squared LD metric. If we let $\mathcal{S}_0 = 2$, if $p_{l_1(1)} = 1$, $p_{l_1(2)} = 0$, and $p_{l_2(1)} = 1$, $p_{l_2(2)} = 0$, even $D_{l_1 l_2} = 0$ it still produces $r^2_{l_1 l_2} = 0.25$, an inflated LD driven by the population structure.

$$
\begin{cases}
m_e &= \frac{m^2}{\sum_{l_1, l_2} r^2_{l_1 l_2}} = \frac{m^2}{m + \sum_{l_1 \neq l_2} r^2_{l_1 l_2}} \\
\tilde{m}_e &= \frac{m^2}{\sum_{l_1, l_2} \tilde{r}^2_{l_1 l_2}} = \frac{m^2}{m + \sum_{l_1 \neq l_2} \tilde{r}^2_{l_1 l_2}}
\end{cases}
\tag{17}
$$

When there is population structure, $m_e \geq \tilde{m}_e$. It is expected to observe strong shrinkage given increasing population structure.

References

[1] Stam, P. The distribution of the fraction of the genome identical by descent in finite random mating populations. *Genetical Research* **10**, 131–155 (1980).

[2] Visscher, P. M. *et al.* Assumption-free estimation of heritability from genome-wide identity-by-descent sharing between full siblings. *PLoS Genetics* **2**, e41 (2006). URL http://www.pubmedcentral.nih.gov/articlerender.fcgi?artid=1413498&tool=pmcentrez&rendertype=abstract.

[3] Hill, W. G. & Weir, B. S. Variation in actual relationship as a consequence of Mendelian sampling and linkage. *Genetics Research* **93**, 47–64 (2011). URL http://www.pubmedcentral.nih.gov/articlerender.fcgi?artid=3070763&tool=pmcentrez&rendertype=abstract.

[4] Goddard, M. Genomic selection: prediction of accuracy and maximisation of long term response. *Genetica* **136**, 245–257 (2009). URL http://www.ncbi.nlm.nih.gov/pubmed/18704696.

[5] Hayes, B. J., Visscher, P. M. & Goddard, M. E. Increased accuracy of artificial selection by using the realized relationship matrix. *Genetics Research* **91**, 47–60 (2009).

[6] Goddard, M. E., Hayes, B. J. & Meuwissen, T. H. Using the genomic relationship matrix to predict the accuracy of genomic selection. *Journal of Animal Breeding and Genetics* **128**, 409–421 (2011).

[7] Purcell, S. M. *et al.* Common polygenic variation contributes to risk of schizophrenia and bipolar disorder. *Nature* **460**, 748–752 (2009). URL http://www.ncbi.nlm.nih.gov/pubmed/19571811.

[8] Dudbridge, F. Power and predictive accuracy of polygenic risk scores. *PLoS Genetics* **9**, e1003348 (2013). URL http://www.pubmedcentral.nih.gov/articlerender.fcgi?artid=3605113&tool=pmcentrez&rendertype=abstract.

[9] Wang, X. *et al.* Polygenic risk prediction: why and when out-of-sample prediction R2 can exceed SNP-based heritability. *American Journal of Human Genetics* **110**, 1207–1215 (2023).

[10] Visscher, P. M. *et al.* Statistical power to detect genetic (co)variance of complex traits using SNP data in unrelated samples. *PLoS Genetics* **10**, e1004269 (2014). URL http://dx.plos.org/10.1371/journal.pgen.1004269.

[11] Chen, G.-B. Estimating heritability of complex traits from genome-wide association studies using IBS-based Haseman-Elston regression. *Frontiers in Genetics* **5**, 107 (2014). URL http://www.frontiersin.org/Statistical_Genetics_and_Methodology/10.3389/fgene.2014.00107/abstract.

[12] Visscher, P. M. & Goddard, M. E. A General Unified Framework To Assess the Sampling Variance of Heritability Estimates Using Pedigree or Marker-Based Relationships. *Genetics* **199**, 223–232 (2015). URL http://www.ncbi.nlm.nih.gov/pubmed/25361897.

[13] Zhou, X. A unified framework for variance component estimation with summary statistics in genome-wide association studies. *Annals of Applied Statistics* **11**, 2027–2051 (2017).

[14] Zhang, Q.-X. *et al.* Searching across-cohort relatives in 54, 092 GWAS samples via encrypted genotype regression. *PLoS Genetics* **20**, e1011037 (2024). URL http://dx.doi.org/10.1371/journal.pgen.1011037.

[15] Speed, D., Hemani, G., Johnson, M. R. & Balding, D. J. Improved Heritability Estimation from Genome-wide SNPs. *American Journal of Human Genetics* **91**, 1011–21 (2012). URL http://www.ncbi.nlm.nih.gov/pubmed/23217325.

[16] Qi, G.-A. *et al.* Analytical and computational solution for the estimation of SNP-heritability in biobank-scale and distributed datasets. *bioRxiv* 614017 (2024).

[17] Hill, W. G. & Robertson, A. The effect of linkage on limits to artificial selection. *Genetical Research* **8**, 269–294 (1966). URL http://www.journals.cambridge.org/abstract_S0016672300010156.

[18] Isserlis, L. On a formula for the product-moment coefficient of any order of a normal frequency distribution in any number of variables. *Biometrika* **12**, 134–139 (1918).

[19] Chang, C. C. *et al.* Second-generation PLINK: rising to the challenge of larger and richer datasets. *GigaScience* **4**, 7 (2015). URL http://gigascience.biomedcentral.com/articles/10.1186/s13742-015-0047-8. 1410.4803.

[20] Theodoris, C., Low, T. M., Pavlidis, P. & Alachiotis, N. quickLD: An efficient software for linkage disequilibrium analyses. *Molecular Ecology Resources* **21**, 2580–2587 (2021).

[21] Wu, Y. & Sankararaman, S. A scalable estimator of SNP heritability for biobank-scale data. *Bioinformatics* **34**, i187–i194 (2018).

[22] Nei, M. & Li, W.-h. LINKAGE DISEQUILIBRIUM IN SUBDIVIDED POPULATIONS. *Genetics* **75**, 213–219 (1973).

[23] Ohta, T. Linkage disequilibrium with the island model. *Genetics* **101**, 139–155 (1982).
